# Characterization of the stress associated microRNAs in *Glycine max *by deep sequencing

**DOI:** 10.1186/1471-2229-11-170

**Published:** 2011-11-23

**Authors:** Haiyan Li, Yuanyuan Dong, Hailong Yin, Nan Wang, Jing Yang, Xiuming Liu, Yanfang Wang, Jinyu Wu, Xiaokun Li

**Affiliations:** 1 Ministry of Education Engineering Research Center of Bioreactor and Pharmaceutical Development, Jilin Agricultural University, Changchun, Jilin 130118, China; 2 College of Life Sciences, Jilin Agricultural University, Changchun, Jilin 130118, China; 3 Institute of Biomedical Informatics, Wenzhou Medical College, Wenzhou 325000, China

## Abstract

**Background:**

Plants involved in highly complex and well-coordinated systems have evolved a considerable degree of developmental plasticity, thus minimizing the damage caused by stress. MicroRNAs (miRNAs) have recently emerged as key regulators in gene regulation, developmental processes and stress tolerance in plants.

**Results:**

In this study, soybean miRNAs associated with stress responses (drought, salinity, and alkalinity) have been identified and analyzed in combination with deep sequencing technology and in-depth bioinformatics analysis. One hundred and thirty three conserved miRNAs representing 95 miRNA families were expressed in soybeans under three treatments. In addition, 71, 50, and 45 miRNAs are either uniquely or differently expressed under drought, salinity, and alkalinity, respectively, suggesting that many miRNAs are inducible and are differentially expressed in response to certain stress.

**Conclusion:**

Our study has important implications for further identification of gene regulation under abiotic stresses and significantly contributes a complete profile of miRNAs in *Glycine max*.

## Background

Terrestrial plants face serious abiotic stresses (e.g. drought, salinity, alkalinity, cold, pathogen responses and diseases), these are the predominant cause of decreased crop yields [1]. Being one of the major oil crops worldwide, *Glycine max *faces these challenges posed by environmental stressors. To cope with environmental stresses, crops have evolved sophisticated adaptive response mechanisms [2]. Therefore, unraveling the complex resistant mechanisms of soybeans will provide fundamental insights into the biological processes involved in environmental stimuli, which may prove helpful in alleviating crop losses.

There is increasing evidence that microRNAs (miRNAs), ~21 nucleotides (nt) in length, act as key factors in gene regulation, developmental processes and stress tolerance in plants [3-5]. MiRNAs function by either cleaving their targets (mRNAs predominantly via RISC) or repressing protein translation [6,7]. Indeed, it has been suggested that a number of miRNAs that participate in stress responses have adapted to environmental challenges. For example, Phillips et al. [8] reported that miR395, miR397b, and miR402 are involved in stress response. Expression levels of miR393 changed under salinity and alkaline stresses, however, over-expression of miR393 is harmful to plants [9]. In response to environmental stresses, fluctuations in the expression of miRNAs can be induced by many uncontrolled factors, such as drought, salinity, and alkalinity at transcriptional and post-transcriptional levels. It was reported that sulfate starvation lead to the up-regulation of miRNA395 [7] miR398 and miR408 were responded to water deficiency [10]. Furthermore, these inducible miRNAs display different specificity under different stresses. However, our knowledge of the roles played by miRNAs under stress conditions in plants is still limited, especially at the whole-genome level.

In recent years, it has been possible to identify miRNAs through either bioinformatics or sequencing. For instance, various methods have been used to identify miRNAs in rice, wheat, and maize [11-13]. Many bioinformatics approaches and technologies have been developed for rapid and accurate miRNA detection and analysis. Recently, deep sequencing technology is showing significant promise for small RNA discovery and genome wide transcriptome analysis at single-base pair resolution [14]. In comparison with microarray, deep sequencing has several advantages, the major one being its application in comprehensively identifying and profiling small RNA populations that were previously unknown. Deep sequencing has identified many small RNAs in different plants, mutants, and tissues at various developmental stages [15-18]. In this study, soybean miRNAs associated with stress response were identified and analyzed by high-throughput sequencing. One hundred and thirty three known miRNAs corresponding to 95 miRNA families were detected in soybeans under three stress treatments. In addition, 71, 50, and 45 miRNAs were differentially expressed under drought, salinity, and alkalinity, respectively, suggesting that many miRNAs are inducible and are differentially expressed during different environmental stresses.

## Results

### General features of small RNA transcriptomes under diverse treatments

**Figure 1:**
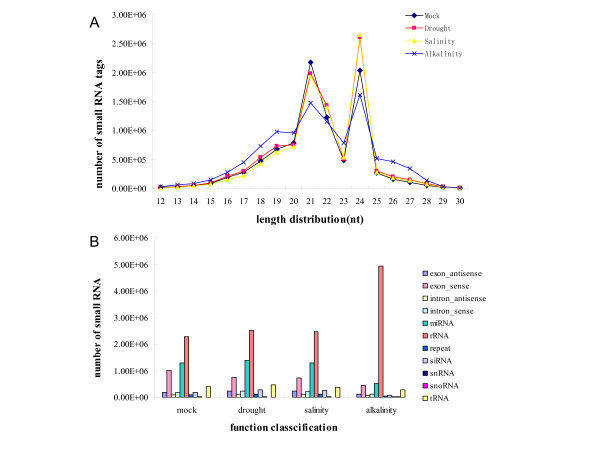
**Length size distribution of small RNA**. The length size distribution (A) and proportions of various categories (B) of small RNAs in soybean under the drought, salinity and alkalinity treatments and mock group.

Small RNAs were documented not only to modulate a series of complex developmental events, but also to regulate defense under abiotic stress [19,20]. To explore the small RNA pools from three stress treatments in soybeans (mock, drought, salinity, and alkalinity), RNA libraries were generated and sequenced by Solexa (Illumina). More than 36 million original sequencing tags were produced with approximately 9-10 million raw reads from each library. After discarding low quality, filtering 5' contaminant and trimming 3' adaptor reads, a total of 8,500,978, 9,357,545, 9,003,582 and 9,223,744 clean reads were obtained from mock, drought, salinity and alkalinity treated datasets, respectively (see Additional file 1). Although the total numbers of sequence reads in four RNA libraries were similar, the size distribution of sequence tags was substantially different (Figure 1A, Additional file 2). For example, 2 182 055 (23.72% of clean reads from mock) sequences are canonical 21 nt small RNAs with the most abundant small RNAs in the roots of mock samples. While 1 982 765, 1 929 505 and 1 476 829 reads of 21 nt were in the three stressed libraries, accounting for 19.64% of clean reads from drought, 20.22% of clean reads from salinity and 14.33% of clean reads from alkalinity, respectively. Small RNAs varied widely in length and redundancy, the 24 nt reads showed the highest redundancies (27.78%) in the salinity induced library. The 24 nt reads constitute 25.90% and 22.14% in drought and mock libraries, while they only account for 15.69% in the alkalinity induced library. The relatively lower percentage of 24 nt reads indicates that more kinds of miRNAs are involved in the response of *G. max *to alkalinity compared with other stress conditions. These data highlight the overall complexity of the small RNA repertoire under different stress conditions.

It is essential to generate a reference set of annotations for exploring the small RNA categories. All identical Solexa reads in each library were sorted into unique sequence tags for further analysis. When aligned, all sequences were read against the *Glycine max *genome using SOAP2 [21], about 70% of reads matched perfectly and 30% were from un-annotated genome sites with one mismatch. For instance, in the mock, 7,045,434 (75.4%) clean reads that grouped into 1,609,063 unique reads were matched to the 1 115 Mb genome of *Glycine max*. Subsequently, for each library approximately 60% of clean small RNAs were identified as products processed from rRNAs, tRNAs, snRNAs, or other non-coding RNAs (Figure 1B). Another fraction (approximately 40%) was predominantly derived from un-annotated or repeated sequences. Large portions of annotated small RNAs were mainly non-coding RNAs. For the mock group, 1 289 824 clean sequences which were classified into 1,474 unique tags were considered to be potential miRNAs. The other two induced by drought and salinity were 1,393,901 (1,512 unique tags) and 1,302,431 (1,503 unique tags), respectively. Notably, in the alkalinity-induced group, 513,021 screened reads (1,062 unique tags) were miRNA candidates, accounting for nearly half of miRNAs of the former three groups. It is estimated that known miRNAs might be the most abundant class of small RNAs regulated at post-transcriptional levels in plant defense.

### Known miRNAs in soybean

Many miRNAs of the soybean have been reported in previous studies. Kulcheski et al. [22] detected 256 miRNAs from drought-sensitive and tolerant seedlings and rust-susceptible and resistant soybeans, of which 24 families of miRNAs had not been reported before. Song et al. [15] identified 26 new miRNAs in developing soybean seeds by deep sequencing. Joshi [23] identified 129 miRNAs based on sequencing and bioinformatic analyses, among which, 42 miRNAs matched known miRNAs in soybean or other species, while 87 were novel miRNAs. In another study Chen et al. [24], reported 15 conserved miRNA candidates belonging to eight different families and nine novel miRNA candidates comprising eight families in wild soybean seedlings. To identify known miRNAs from the soybean in four diverse treatments, small RNA sequences were compared with miRBase 16.0. After a sequence similarity search, 133 known miRNAs corresponding to 95 miRNA families were identified in the soybean (Additional file 3). In addition, four conserved star miRNAs (miR156d*, miR157b*, miR162*, and miR3630*) have also been sequenced. Among them, miR156d*, miR157b*, and miR3630* star sequence expressions were rather low. However, the abundance of miR162* ranged from 125 to 220 reads under different treatments. In addition, other star miRNAs expression levels were low under all four conditions, these were miR172b*, miR156h*, and miR166g*. Other studies showed that miRNAs are often evolutionarily conserved throughout the plants [25,26]. Hence, we investigated the evolutionary conservation features of the identified miRNAs in soybean by comparing them to *Arabidopsis thaliana*, *rice*, *Zea mays*, *Medicago truncatula*, *Sorghum bicolor*, *Triticum aestivum*, *Vitis vinifera*, *brassica *and *Pinus *according to their sequence similarity (data not shown). The identified miRNA families are conserved in a variety of plant species. One hundred and ten miRNA genes were reported in *Glycine max*, the other 23 genes were detected from known orthologous miRNAs.

**Figure 2:**
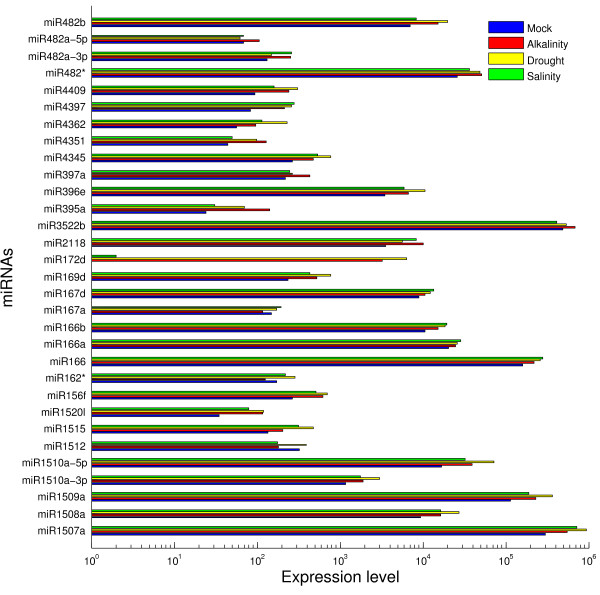
**Most abundantly expressed miRNA**. The most abundantly expressed miRNAs under three stresses and matched mock group.

**Figure 3:**
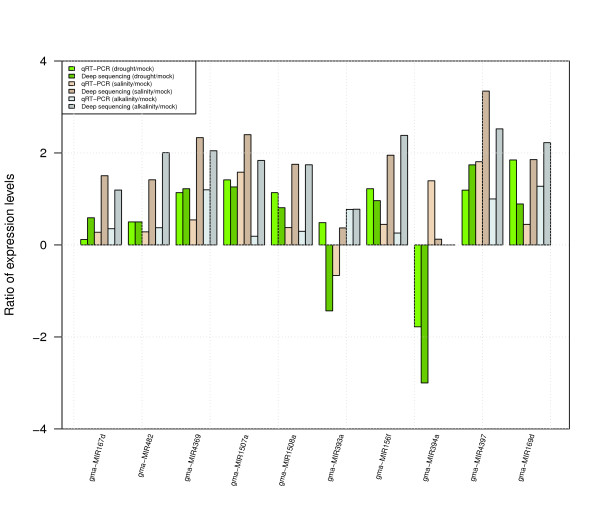
**Expression pattern of miRNA**. miRNA expression levels from Solexa sequencing and qRT-PCR. Expression pattern of the selected 10 known miRNAs (miR156f, miR167d, miR169d, miR393a, miR394a, miR482, miR1507a, miR1508b, miR4369, and miR4397) measured by Solexa and qRT-PCR. 5s small RNA was used as a control in qRT-PCR. Total RNA (1 μl) from each of the four conditions (mock, drought, salinity and alkalinity) were used for verification.

The sequencing frequencies for miRNAs in our four libraries were used as an index for estimating the relative abundance of 133 miRNAs. The distribution patterns of miRNA frequencies varied greatly, indicating that these miRNAs were expressed ubiquitously in each library. Three abundant miRNA reads (miR166, miR1507, and miR3522) occupied 79.47% of expressed miRNA tags on average (Figure 2, Additional file 4 and Additional file 5). The identified miRNA families are conserved in a variety of plant species in our study. For example, families of miR156, miR1507, and miR3522 are widely conserved in 10, 3, and 1 species, respectively (see Additional file 6). Most mature miRNAs identified in the soybean were also detected in other plant species, such as *Arabidopsis *[27], grapevine [28], and poplar [29]. Especially those present in high abundance, such as miR156, miR166, and miR167. Of these, miR166 was the most abundant (with sequence reads of 263 470 times under drought). Previous studies revealed that miRNAs with high expression levels always correlate with evolutionary conservation [25,30]. In this study, the majority of miRNAs occurring at low frequencies, with no more than 100 read tags, such as miR408 and miR1517, showed poor conservation. Nevertheless, the miRNAs with the least sequence reads, including miR169g, miR171b, and miR393b, were sequenced dozens of times but were conserved in 9, 17 and 8 plant species, respectively (Figure 3). MiR171b expressed in the mock and miR393b expressed in drought were sequenced 21 and 0 times, respectively. These observations suggest that conserved miRNAs may be essential for controlling basic cellular and developmental pathways (e.g. cell cycle) in plants.

To validate the expression pattern of miRNAs by deep sequencing, we randomly selected ten miRNAs (miR156f, miR167d, miR169d, miR393a, miR394a, miR482, miR1507a, miR1508b, miR4369, and miR4397) to perform verification by qRT-PCR. Expression abundance patterns in three stress (drought, salinity, and alkalinity) induced samples were compared with the mock. Up-regulated miRNAs under three stress-induced conditions, which occurred most frequently with both methods, were miR167d, miR169d, miR482, miR1507a, miR1508b and miR4369. Only miR393a had shown to be not in accordance with Solexa result. MiR394a was down-regulated and exhibited an identical pattern in both methods. These highly concordant results between two methods suggest qRT-PCR validation indicated a good concordance of both methods (Figure 3).

### Novel miRNAs in soybean

From the four small RNA libraries, 102 miRNAs were revealed as possible miRNA candidates of soybean. To support the existence of the novel miRNAs, their hairpin structures and free energies were used to evaluate these candidate miRNAs. We identified 50 novel miRNAs, with the 10 most highly expressed candidates listed in Table 1, and the others in Additional file 7. The energy scope of these miRNAs ranged from 70.8 kcal/mol (Gma-050) to -24.2 kcal/mol (Gma-013). The expression levels of these candidates ranged broadly, from thousands of sequence counts to single sequence counts. Most mature sequences were products of a step-loop structure at both 5' and 3' mediated by Dicer-like enzymes. Novel miRNAs, including Gma-m0004, Gma-m008, Gma-m009, Gma-m011, and Gma-m030, were identified at both the 3' and 5' ends of hairpins. The 5' read tags displayed very small read counts compared with 3' tags. Gma-m045, Gma-m046, Gma-m030, and Gma-m050 showed nearly equal numbers of sequence reads originating from both arms of the miRNA precursors. Eleven miRNAs, including Gma-m006, had a higher number of sequence reads originating from the 5' arm than the annotated mature miRNA containing 3' arm, suggesting that the majority of miRNA genes processed by DCL have a strand bias in plants.

**Table 1:** The top 10 novel miRNAs predicted from both arms of the miRNA precursor

miRID	Location	Strand (+/-)	Energy (kcal/mol)	Sequence of 5p	Sequence of 3p	Mock (count)		Drought (count)		Salinity (count)		Alkalinity (count)	
						
						5p	3p	5p	3p	5p	3p	5p	3p
Gma-m001	Gm18:61442586:61442692	-	-44.1	CTGACAGAAGATAGAGAGCAC	-	3395	-	3248	-	4830	-	2216	-
Gma-m002	Gm02:837420:837549	+	-56.5	CAGGGGAACAGGCAGAGCATG	-	3672	-	2573	-	2945	-	3159	-
Gma-m003	Gm12:3176108:3176377	+	-69.67	TCCATTGTCGTCCAGCGGTTA	-	3282	-	3673	-	2931	-	1186	-
Gma-m004	Gm19:40699070:40699221	-	-65.8	TGGGTGAGAGAAACGCGTATC	TACGGGTCGCTCTCACCTAGG	367	879	491	1053	186	1083	125	329
Gma-m005	Gm14:5324794:5324912	+	-44	AGCCAAGAATGACTTGCCGGAA	CGGGCAAGTTGTTTTTGGCTAC	337	560	475	644	438	471	175	173
Gma-m006	Gm09:16565920:16566038	-	-44.7	AGAGGTGTTTGGGATGAGAGA	CCTCATTCCAAACATCATCTAA	1596	102	1695	138	777	128	335	53
Gma-m007	Gm18:61452908:61452997	-	-41	GGAATGGGCTGATTGGGAAGT	-	835	-	781	-	813	-	598	-
Gma-m008	Gm02:30498945:30499130	-	-70.5	CTGGGTGAGAGAAACACGTAT	ACGGGTCGCTCTCACCTGGAG	85	665	170	635	78	714	43	264
Gma-m009	Gm13:34382988:34383131	-	-57.37	TCATTGAGTGCAGCGTTGATG	TATTGACGCTGCACTCAATCA	332	811	187	744	202	340	142	227
Gma-m010	Gm06:10859290:10859391	-	-33.76	-	CGAGCCGAATCAATACCACTC	-	658	-	693	-	515	-	355

In comparison with these conserved miRNAs, all the novel miRNA tags had low read counts in the four libraries, where the highest is only 4 830 at 5' end (Gma-001). The least is only one at 3' and 5' end (e.g. Gma-011, Gma-023, Gma-025, Gma-026, Gma-037, Gma-039, Gma-040, Gma-047), and the average read count was 318. It is well known that conserved miRNAs are highly expressed frequently and ubiquitously whereas non-conserved miRNAs are not. Further experimentation is needed to determine whether these novel miRNAs are stress induced.

### MiRNAs expression patterns under drought, salinity, and alkalinity

**Figure 4:**
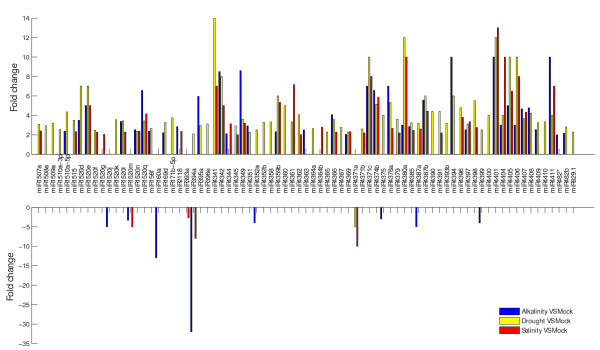
**Different expressed miRNAs**. The most significantly different expressed miRNAs under the three stresses of drought, salinity, and alkalinity in comparison with that of the mock.

To gain deep insight into environmental adaptation of soybean, we studied common and unique miRNA expression patterns under drought, salinity, and alkalinity conditions. As shown in Figure 4, miRNA expression varied in response to different stress-inducing conditions. These genes were identified as functional regulation factors in the resistance of stress. The miRNA expression profiles observed revealed that a small portion of miRNAs (miR434a, miR157b*, and miR171a) exhibited stress-specific expression patterns. Moreover, all of the three miRNAs have low expression abundance. Substantial portions of the miRNAs were expressed under two or three stress conditions. For example, miR156d*, miR160a, miR394a, miR1520j, miR4341, miR4387a, miR4399, miR1520c, and miR1520r appeared in three stress conditions while miR169g, miR1517, and miR3630* appeared in two stress conditions. Therefore, some miRNA expressing intermediate counts (e.g. miR160a and miR394a) and others had only several reads (e.g. miR-156d*, miR169g, and miR393b).

The vast majority of the differentially expressed miRNAs showed different expression patterns either among three conditions or between two stress conditions. Of these, the expression of 78 miRNAs was significantly different (fold change > 2; p < 0.05) (Figure 4), these were congruously or differentially regulated under the three stress conditions. In three stress conditions, 27 miRNAs (e.g. miR1520d, miR1520n, and miR4407) were all up-regulated in comparison to the mock. For example, the expression level of miR4407 changed 3.67, 4.33, and 4.67 folds in drought, salinity, and alkalinity, respectively. Fifty-one miRNAs showed different trends under various inducing conditions (such as miR394a, miR4361, miR4396, and miR4308), indicating that individual miRNAs may have distinctive expression patterns under different stress conditions. For example, miR394a was up-regulated in drought (fold change = 2.09) but down-regulated in salinity (fold change = -8). Under different conditions, 70, 46 and 37 miRNAs were up-regulated with a fold change > 2 (e.g. miR169d), and 1, 4 and 8 were down-regulated with fold changes > -2 (e.g. miR393a) in drought, salinity and alkalinity, respectively. The expression profiles strongly indicate that different miRNA regulation patterns might completely or partly contribute to explaining the stress regulation between various treatments.

### MiRNA targets prediction

**Figure 5:**
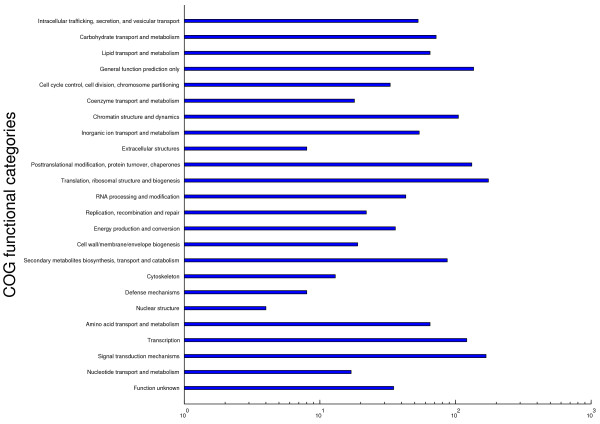
**COG functional classification**. COG functional classification of the predicted target genes of identified miRNAs in soybean.

Investigation of the target mRNAs of the miRNAs identified can assist us in understanding their biological roles [31,32]. In a previous study, Katara et al. [33], predicted 573 targets for 44 of the 69 mature miRNA sequences published in the database. Study of affected proteins revealed that more of the target protein products were involved in diverse physiological processes e.g. photosynthesis [34]. Joshi [23] predicted the putative target genes of 129 identified miRNAs with computational methods and verified the predicted cleavage sites in vivo for a subset of these targets using the 5' RACE method. In addition, the authors also studied the relationship between the abundance of miRNA and that of the respective target genes by comparing their results to Solexa cDNA sequencing data. In the study of Song et al. [15], 145 were identified as targets of 38 known miRNAs and 8 new miRNAs and 25 genes. GO analysis indicated that many of the identified miRNA targets may function in soybean seed development To understand the relationship between the soybean the miRNAs identified in the four treatments with previously published mRNAs, we utilized the psRNATarget program for predicting mRNA targets of miRNAs. 1 219 mRNAs were predicted to be targets for 126 miRNAs (Additional file 8, Additional file 9). Finally, 989 genes were classified into 24 types annotated by COG (Figure 5). The function of most mRNAs is translation, ribosomal structure and biogenesis, and signal transduction mechanisms. Furthermore, a variety of biological functions are involved in nucleotide transport and metabolism, transcription, defense mechanisms etc, which will provide useful information about the regulatory roles of miRNAs for different tolerances. These results demonstrate that the majority of targets fall into the category of transcriptional regulation, indicating that these targets encode transcription factors (e.g. target of miR169d: CBF-B/NF-Y transcriptional factor). Some miRNAs, such as gma-miR156f and gma-miR172d, have multiple target sites, indicating that these miRNAs are functionally divergent. Additionally, a single gene may be targeted by several miRNAs, such as polyphenol oxidase, which is regulated by gma-miR157b and gma-miR3522b.

### Mature miRNA quantification by northern blotting

**Figure 6:**
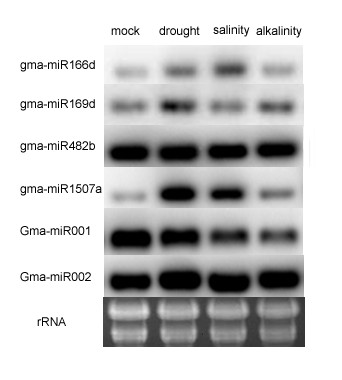
**Northern blotting**. Northern blotting confirming differential expression of miRNAs. Total RNA (30 μg) from each of the four conditions (mock, drought, salinity, and alkalinity) was loaded and probed with miRNAs probe. The blot was hybridized with six miRNAs (miR166b, miR169d, miR482b, miR1507a, Gma-m001, and Gma-m002). The rRNA bands were shown as a loading control. miR166b, miR169d, and miR1507a expressed identical patterns when compared with Solexa sequencing.

To confirm and validate the results obtained from the Solexa library, we examined the expression patterns of four known miRNAs and two novel miRNAs. These six miRNAs (miR166b, miR169d, miR482b, miR1507a, Gma-m001, and Gma-m002) were individually selected and experimentally verified by northern blotting hybridization. The sequences of antisense RNA probes are listed in Additional file 10. By comparing the miRNA results by Solexa sequencing to northern hybridization, three stress-responsive miRNAs (miR166b, miR169d, and miR1507a) were identified with identical expression patterns. MiR166b and MiR1507a were up-regulated under drought, salinity, and alkalinity conditions. MiR169d was up-regulated under drought and alkalinity (Figure 6). While the expression patterns of miR482b and Gma-m002 remained unchanged by the three stress conditions when tested by northern blotting. However, these were up-regulated under drought stress according to the Solexa results. Based on the northern blot analysis, the expression level of Gma-m001 decreased under salinity stress, but identical patterns were observed under drought and alkalinity when compared with Solexa sequencing (Figure 6). Therefore, the expression pattern obtained by RNA blot analysis may reflect the result from deep sequencing.

## Discussion

Nowadays, characterization of the vital roles of miRNAs play in plant stress responses is an active research field. Although many studies have demonstrated that plant miRNAs function as important regulators in development and morphogenesis processes, more reports are indicating that plant miRNAs are also involved in environmental stress tolerance [7].

Since abiotic stress is one of the primary causes of crop losses worldwide, unraveling the complex mechanisms underlying stress resistance of plants has profound significance. Recently, the newly developed sequencing technologies, such as the Illumina Genome Analyzer (GA), Roche/454 FLX system, and the ABI SOLiD system, show advances over traditional methods with improved throughput and dramatically reduced cost. Currently, applications of high-throughput sequencing technologies are arousing much research interest, such as identification of entire sets of miRNAs, which deliver new insights into the role of miRNAs in plant development, and stress related regulation. By using this method, a number of soybean miRNAs have been well annotated [34]. Differing from microarray, high throughput sequencing allows us to comprehensively survey stress related miRNAs. To date, little is known about the functions of miRNAs in abiotic stress responses in *Glycine max*.

In this study, we sequenced and analyzed small RNAs of the soybean under three treatments based on deep sequencing. Investigation of the small RNAs showed that gma-miR1507a (936 627 sequence tags) was represented in our sequencing libraries. One hundred and thirty three known miRNAs and 50 novel miRNAs were obtained from next generation sequencing data. Through expression abundance of the miRNA repertoires under drought, salinity, and alkalinity stress conditions, many miRNAs were found to have a wide range of expression levels between libraries. This characteristic of variability in miRNA expression may be due to miRNA mature processing [35], and/or stress associated regulation [2,36]. We envision that these miRNAs might have functional significance, suggesting they may participate in the plant stress response. Highly abundant miRNAs seem to exhibit similar conservation. For example, miR2188 and miR3522b exhibit high expression levels in all four libraries and are conserved across many species. Such observations support previous results that the most abundant miRNAs were phylogenetically conservative [37].

Both miRNAs and star miRNAs are generated from step-loop hairpin structures. MiRNAs are stable and participate in translational repression or cleavage of mRNA by binding or anchoring to the coding region of mRNA sequences [4]. Khvorova et al. [38] inferred from the considerably low abundance of star miRNAs that these strands are typically destroyed when released from pre-miRNA stem. The low expression levels of star miRNA sequences, such as miR156d*, miR157b*, and miR3630*, further support the miRNA synthesis hypothesis. Next generation sequencing is a powerful tool in the detection of miRNA and star miRNA [[15,39] and [40]]. The correlation between star miRNA and its flexible expression may reveal its particular regulated function. MiR162* and miR482* may be involved in regulating stress. Two arms of a single hairpin, giving rise to RNA function isolation by different sequences, may associate with distinct biological activities. Small novel miRNAs annotated in our study, such as the 5' and 3' of Gma-004 and Gma006, were derived from predicted hairpin structures.

Plant miRNAs have been reported as having a strong propensity towards regulating responses to abiotic stress, including dehydration, freezing, salinity, alkalinity, and other stresses by transcriptional factors or proteins [7]. Expression levels of miRNAs induced by environmental stressors vary. They therefore may play a key role in targeting stress-regulated genes. It has been reported that stress response miRNAs were ubiquitously present in *Populus *[41], soybean [22], and other plants. Previous studies have reported that members of miR167, miR319, and miR393 were similarly regulated in stress tolerance [9,42,43]. In this study, members of miR1520n, miR4374b, and miR4396 were up-regulated simultaneously under three stresses, which implies that they might target genes that function as negative regulators of stress tolerance. In addition, it was previously reported that miR395 was previously reported to be up-regulated in a salt induced soybean line targeting sulfurylase and ASP1 genes under sulfate starvation conditions. Therefore, we speculate that miR395 might be involved in non-specific salt-induced responding pathways, such as the maintenance of energy supply [7,13]. Moreover, miR166 is responsive to dehydration stress in barley [44], and it is abundant and up-regulated in soybean seedlings under dehydration conditions. MiR393a, targeting F-box proteins and a basic-helix-loop-helix family protein, was up-regulated in cold, dehydration, salt, or ABA stress [7], and down-regulated in soybean under alkaline stress. These responsive miRNAs are involved in post-transcriptional regulation during stress responsive processes.

Deep sequencing of the small RNA transcriptome yields an incredible amount of data, from which we can not only determine known miRNAs, but also successfully explore novel miRNAs with high accuracy and efficiency. First, in this study, we have identified 133 known and 50 novel miRNAs in *Glycine max*, which illustrates the diversity of miRNA expression in *Glycine max*, revealing the presence of more miRNAs than previously known. In addition, deep sequencing technologies in combination with bioinformatics analysis enabled us to profile the miRNA expression patterns for further miRNA functional insights, and to elucidate the underlying molecular mechanisms and diverse physiological pathways. Second, comparing miRNA expression profiles under various induced conditions, we found significant differences in miRNA regulation patterns, with 71, 50, and 45 altered expression patterns under drought, salinity, and alkalinity, respectively. The differentially expressed miRNAs obtained in this study can serve as a basis for further identification of the regulation roles of stress tolerance in *Glycine max*.

## Conclusion

In this study, soybean miRNAs associated with stress responses (drought, salinity, and alkalinity) have been identified and analyzed in combination with deep sequencing technology and in-depth bioinformatics analysis. One hundred and thirty three conserved miRNAs representing 95 miRNA families were expressed in soybeans under three treatments. In addition, 71, 50, and 45 miRNAs are either uniquely or differently expressed under drought, salinity, and alkalinity, respectively, suggesting that many miRNAs are inducible and are differentially expressed in response to certain stress. This study has important implications for further identification of gene regulation under abiotic stresses and significantly contributes a complete profile of miRNAs in *Glycine max*.

## Materials and methods

### Sample collection and treatment

An inbred line of 'HJ-1', one of the abiotic stress sensitive soybeans, was used in our study. For each inbred line, the uniform seeds were treated with ethanol for 10 minutes and then rinsed several times with sterile distilled water. These seeds were cultured in 1 × Hoagland's nutrient solution (4 ml/L Fe-sequestrene, 6 mM K+ and 4 mM Ca2+). When the four leaf stage was reached, we began to put them under different stress treatments salt (120 mM NaCl), alkalinity (70 mM NaCl and 50 mM NaHCO_3_) and drought (2% PEG) stress) for 48 hours, with the unstressed plants as a mock. Then roots of 120 seedlings were collected and frozen in liquid nitrogen for later use.

### Small RNA sequencing library construction

The isolated RNA samples were purified on 15% PAGE gel for size selection. Small RNAs, < 30 bases, were ligated with a pair of Solexa sequencing adaptor primers (5'-pUCGUAUGCCGUCUUCUGCUUGidT-3' and 5'-GUUCAGAGUUCUACAGUCCGACGAUC-3') using T4 RNA ligase. Ligated RNA was size-fractionated on a 10% agarose gel and the 70-90 nt fractions were amplified for 15 cycles to transform RNA to cDNA to produce sequencing libraries. The purified libraries with approximately 20 mg of small RNA were used for cluster generation and sequencing analysis using the Solexa sequencer (Illumina, San Diego, CA, USA) according to the manufacturer's instructions. All the short reads were deposited in the National Center for Biotechnology Information (NCBI) and can be accessed in the Short Read Archive (SRA) under the accession number SRA045367.1.

### Bioinformatics analysis

After Solexa sequencing, high-quality small RNA reads were extracted from raw reads through filtering out the low quality tags and eliminating contamination of adaptor sequences. The resulted set of unique sequences with related read counts were deemed as clean sequence tags. Matched sequences were then queried against non-coding RNAs (rRNA, tRNA, snRNA, and snoRNA) from Rfam database using SOAP 2.0 program http://soap.genomics.org.cn/. Any small RNA read matches to these sequences were excluded from further analysis. Next, we aligned all sequences against the miRBase16.0 again http://mirbase.org/ using SOAP 2.0 to search for known miRNAs with allowed mismatches (or > 90% identity). To compare miRNA expression data under the four diverse treatments, initially, each identified miRNA read count was normalized to the total number of reads in each given sample. Then, Bayesian method was applied to evaluate the statistical significance (P value). After the Bayesian test, if the P value ≤ 0.01 and the normalized sequence counts changed more than two folds, the specific miRNA was considered to be differently expressed.

Reads that did not match any databases above were marked as unannotated. To identify novel miRNA prediction, small RNA tags that matched miRBase and Rfam were filtered and the remaining tags were aligned with the *Glycine max *genome. To analyze whether the matched sequence could form a suitable hairpin (the secondary structure of the small RNA precursor), sequences surrounding the matched sequence were extracted. The second structure was predicted by RNAfold http://rna.tbi.univie.ac.at/cgi-bin/RNAfold.cgi. Thereafter, novel miRNAs were identified using the MIREAP program developed by the BGI (Beijing Genome Institute, http://sourceforge.net/projects/mireap/) and mirTools [45]. The miRNA candidate targets were predicted using psRNATarget http://bioinfo3.noble.org/psRNATarget/. The COG (Clusters of Orthologous Groups) terms of target genes were annotated by comparing with COGs from NCBI http://www.ncbi.nlm.nih.gov/COG.

### MiRNA quantification by real-time PCR

Total RNA (1 μl) was used for synthetizing reverse transcripts with One Step PrimeScript^® ^miRNA cDNA Synthesis Kit (Takara, Japan) in 20 μl reaction mixture. The reaction was performed at 37°C for 60 min, 85°C for 5 seconds following the manufacturer's instruction. RT-PCR was performed with SYBR^® ^Premix Ex Taq II™ (Takara, Japan). Primers designed in Additional file 11 were used to amplify specific miRNA. Soybean 5s rRNA was used as the endogenous control. Uni-miR qPCR Primer was added as the common reverse primer. The qRT-PCR reactions were carried out in a final volume of 25 μl containing 12.5 μl SYBR^® ^Premix EX Taq™, 1 μl forward and reverse primers, and 2 μl template. To estimate the relative abundance of miRNAs in stress induced samples, the Ct value was directly compared and transformed into a fold-change difference. These reactions were performed using the ABI7300 (Applied Biosystems 7300 Real-Time PCR System).

### MiRNA verification by northern blot

For miRNAs quantification, northern blot hybridization was conducted using the High Sensitive MiRNA Northern Blot Assay Kit (Signosis, USA). 30 μg total RNA of each sample was electrophoresed on a 15% polyacrylamide gel and transferred to membrane (supplied by Signosis). Antisense RNA biotin labeled in the 5' end (Invitrogen, China) was used for hybridization probes. The Cyber Green^® ^II-stained (Biotech, China) rRNA bands in the polyacrylamide gel are shown as a loading control.

## Competing interests

The authors declare that they have no competing interests.

## Authors' contributions

YYD and JYW performed data analysis. HYL, JYW, and YYD wrote the manuscript. HYL and XKL conceived the study. HLY participated in the northern blot verification. NW and JY prepared the samples. XML and YFW participated in the qPCR verification. All the authors approved the final manuscript.

## Supplementary Material

Additional file 1 **Reads abundance of various classification of small RNAs**. Reads abundance of various classification of small RNAs in mock and three stresses, drought, salinity, and alkalinity. Click here for file

Additional file 2 **The length size distribution of small RNAs**. The length size distribution of small RNAs in mock, drought, salinity, and alkalinity, respectively. Click here for file

Additional file 3 **Known miRNAs identified in *Glycine max***. 133 known miRNAs corresponding to 95 miRNA families were identified in sequencing libraries of *Glycine max *under mock and three stresses. Click here for file

Additional file 4 **Frequency distribution of miRNA reads**. Abundant miRNA reads frequency distributed in in mock and three stresses, drought, salinity, and alkalinity. Click here for file

Additional file 5 **Distribution of expressed miRNA tags**. Number of expressed miRNA tags distributed in mock and three stresses, drought, salinity, and alkalinity. Click here for file

Additional file 6 **conserved miRNAs distributed in other species**. miRNA sequences of soybean were conserved in other species. Click here for file

Additional file 7 **Prediction of novel miRNAs**. Novel miRNAs identified from soybean. Click here for file

Additional file 8 **The predicted miRNA targeted genes in *Glycine max***. Click here for file

Additional file 9 **Novel miRNA targeted genes prediction in *Glycine max***. Click here for file

Additional file 10 **The sequences of antisense RNA probes**. Click here for file

Additional file 11 **qRT-PCR primers of miRNAs**. Click here for file
