# Supplementary material for: Characterization of the stress associated microRNAs in Glycine max by deep sequencing
Source: BMC Plant Biol. 2011 Nov 23;11:170. doi: 10.1186/1471-2229-11-170 (PMC3267681; doi:10.1186/1471-2229-11-170)
Supplement: Additional file 1 — Reads abundance of various classification of small RNAs. Reads abundance of various classification of small RNAs in mock and three stresses, drought, salinity, and alkalinity. [file 1471-2229-11-170-S1.DOC]

Additional file 1: Reads abundance of various classification of small RNAs

|  | **mock** | | **drought** | | **salinity** | | **alkalinity** | |
| --- | --- | --- | --- | --- | --- | --- | --- | --- |
| Total | 9344149 | 100% | 10203440 | 100% | 9640581 | 100% | 10392523 | 100% |
| adaptor3_null | 19926 | 0.21% | 14897 | 0.15% | 14101 | 0.15% | 24298 | 0.23% |
| insert_null | 47009 | 0.50% | 16843 | 0.17% | 26166 | 0.27% | 15427 | 0.15% |
| adaptor5_contaminants | 74655 | 0.80% | 77000 | 0.75% | 54639 | 0.57% | 47460 | 0.46% |
| smaller_than_18nt | 700994 | 7.50% | 736507 | 7.22% | 541477 | 5.62% | 1081338 | 10.40% |
| polyA | 587 | 0.01% | 648 | 0.01% | 616 | 0.01% | 256 | 0.00% |
| clean_reads | 8500978 | 90.98% | 9357545 | 91.71% | 9003582 | 93.39% | 9223744 | 88.75% |
