# Supplementary figures and images for: Characterization of the stress associated microRNAs in Glycine max by deep sequencing
Source: BMC Plant Biol. 2011 Nov 23;11:170. doi: 10.1186/1471-2229-11-170 (PMC3267681; doi:10.1186/1471-2229-11-170)

Length Distribution

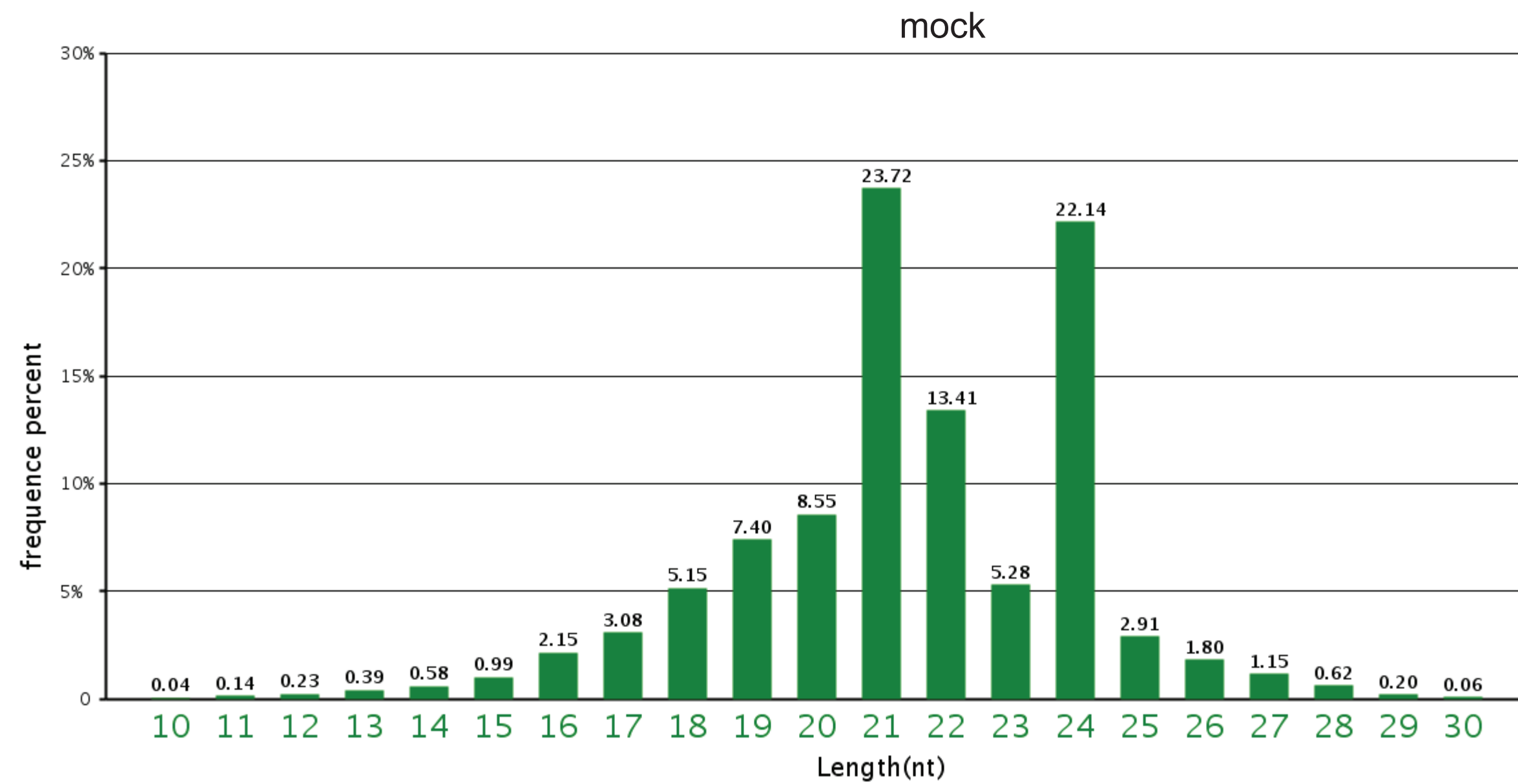

Length Distribution

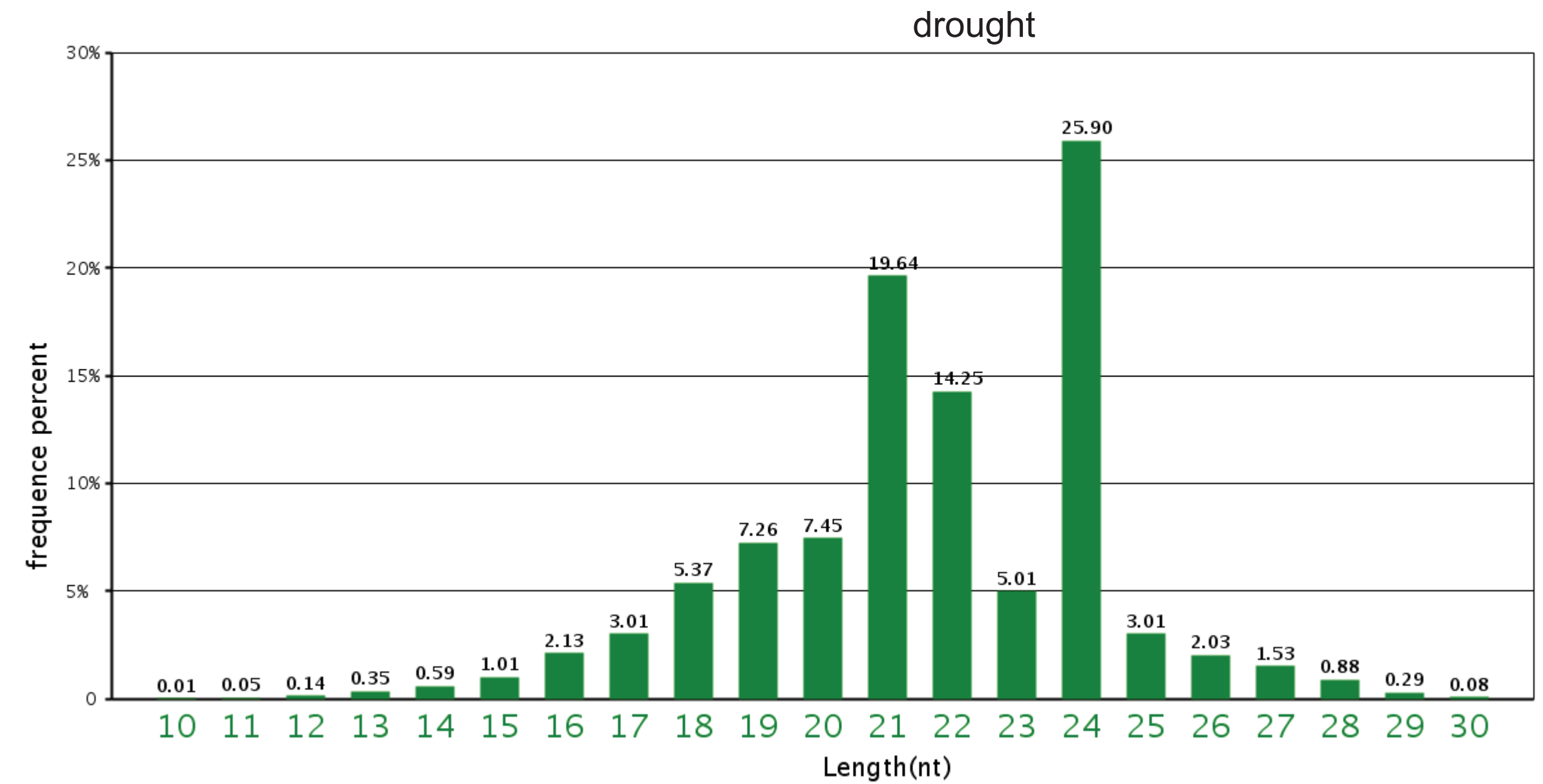

Length Distribution

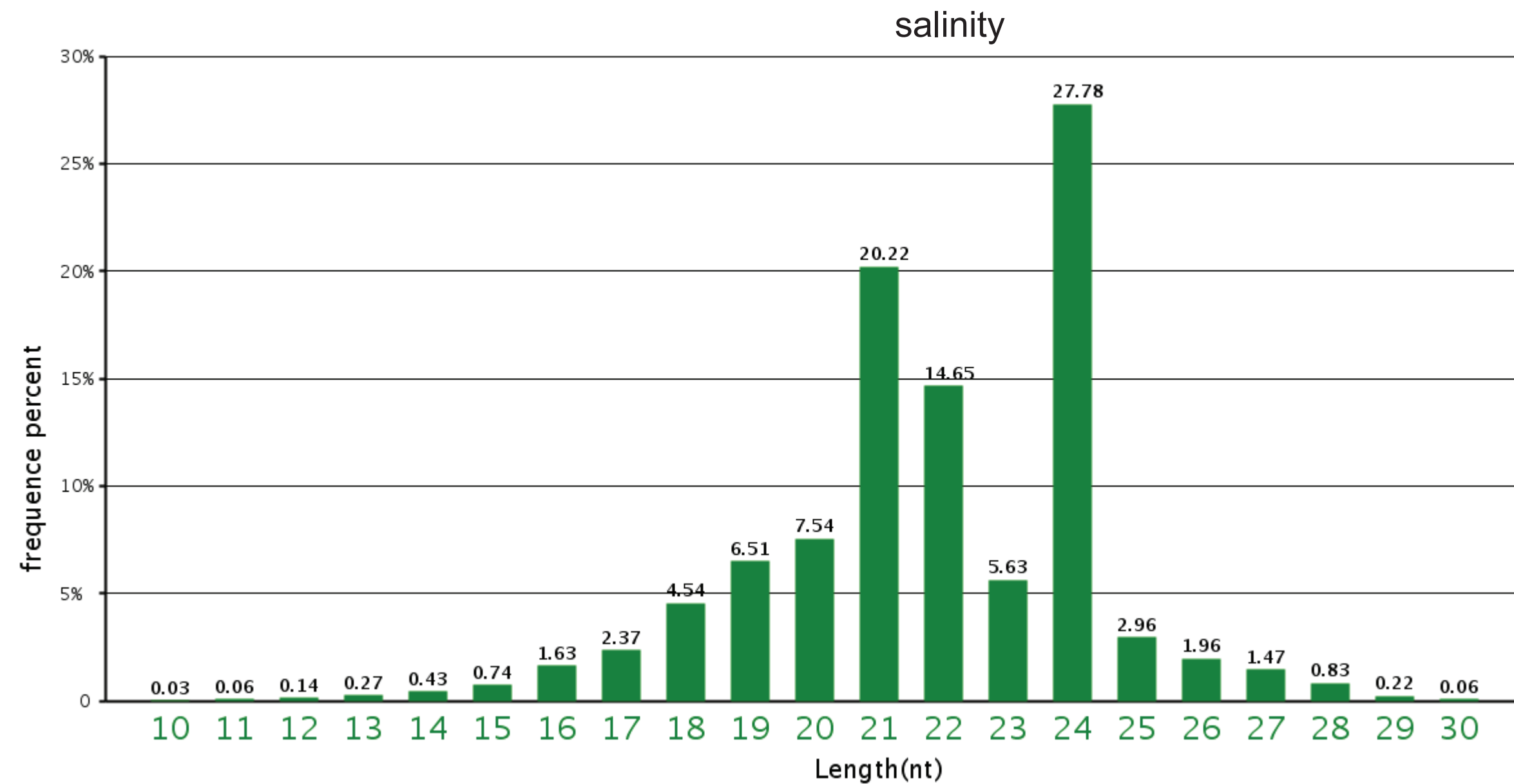

Length Distribution

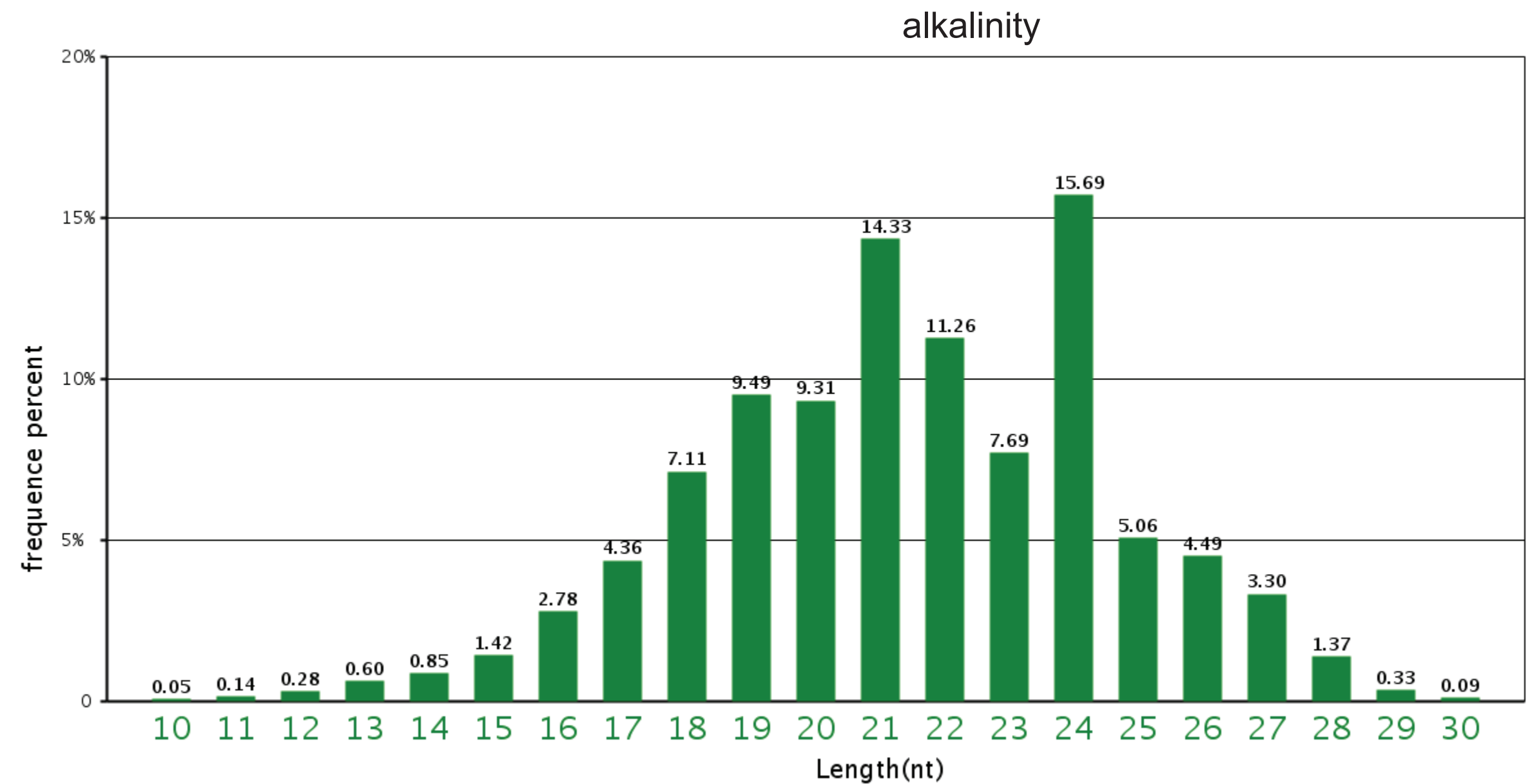

Supplement: Additional file 2 — The length size distribution of small RNAs. The length size distribution of small RNAs in mock, drought, salinity, and alkalinity, respectively. [file 1471-2229-11-170-S2.PDF]

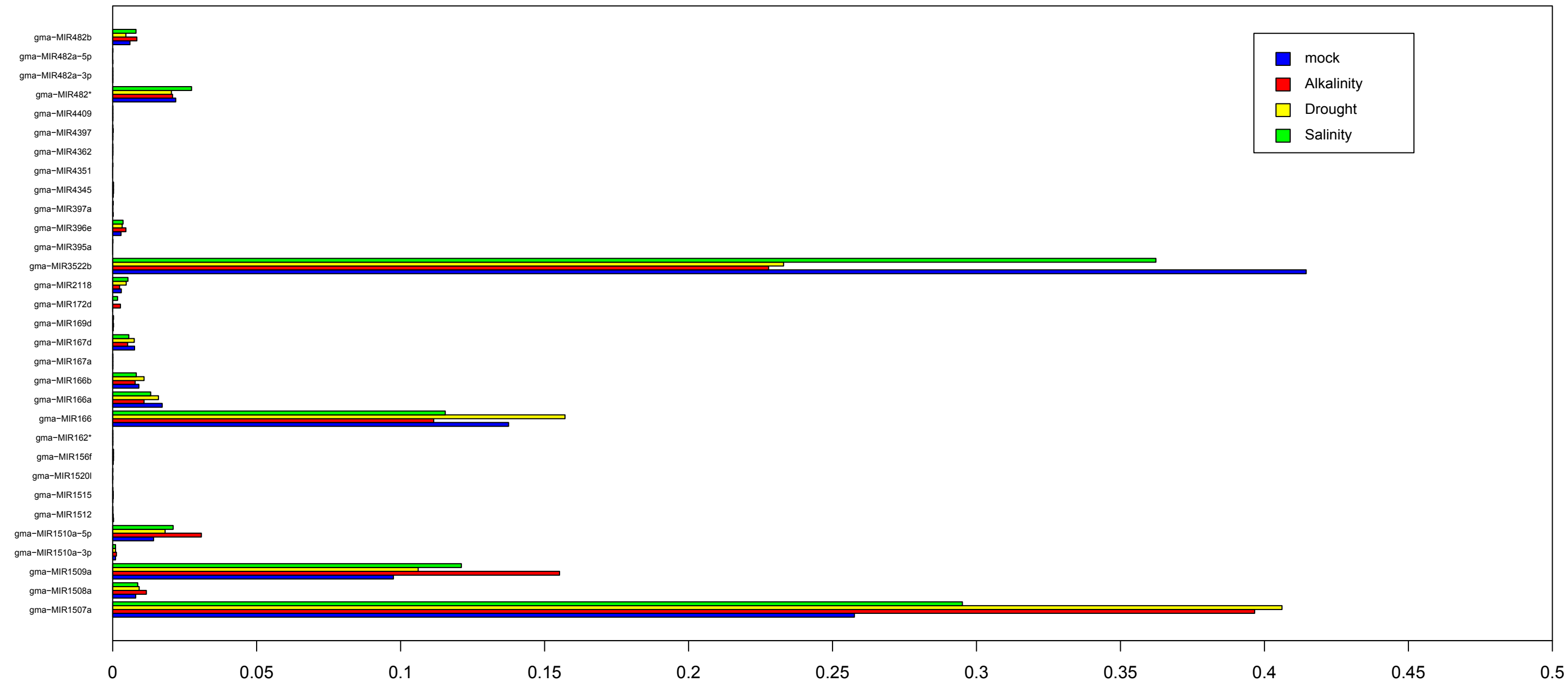

Supplement: Additional file 4 — Frequency distribution of miRNA reads. Abundant miRNA reads frequency distributed in in mock and three stresses, drought, salinity, and alkalinity. [file 1471-2229-11-170-S4.PDF]

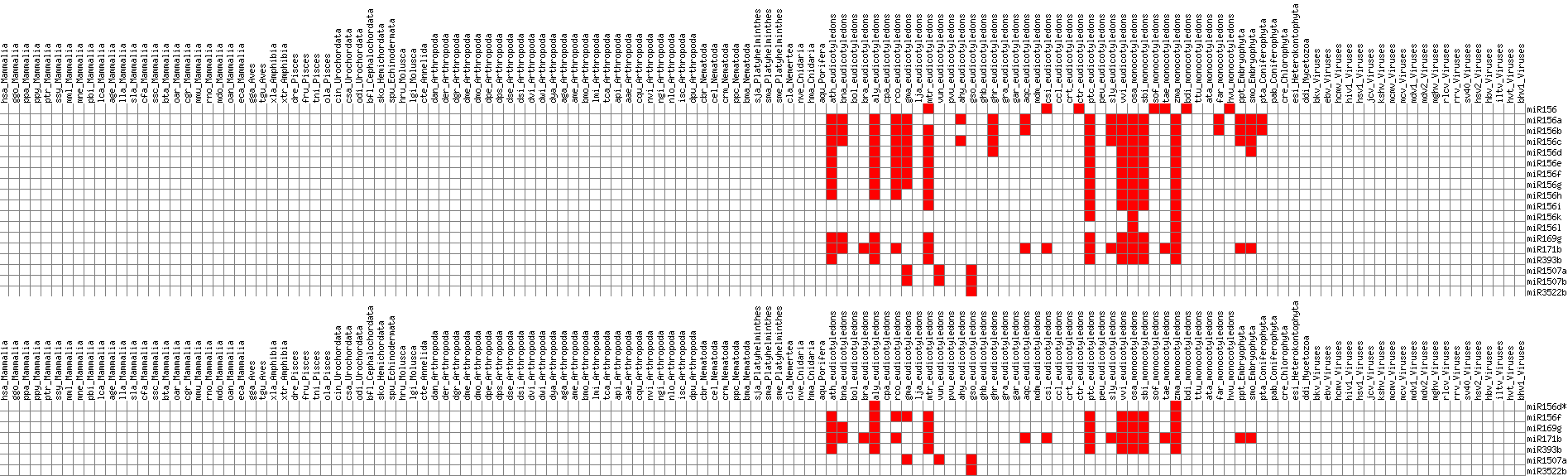

Supplement: Additional file 6 — conserved miRNAs distributed in other species. miRNA sequences of soybean were conserved in other species. [file 1471-2229-11-170-S6.PNG]
