# Supplementary material for: Characterization of the stress associated microRNAs in Glycine max by deep sequencing
Source: BMC Plant Biol. 2011 Nov 23;11:170. doi: 10.1186/1471-2229-11-170 (PMC3267681; doi:10.1186/1471-2229-11-170)
Supplement: Additional file 3 — Known miRNAs identified in Glycine max. 133 known miRNAs corresponding to 95 miRNA families were identified in sequencing libraries of Glycine max under mock and three stresses. [file 1471-2229-11-170-S3.DOC]

**Additional file 3:** Known miRNAs identified in *Glycine max*

| **Family** | **Name** | **Sequence (5'-3')** | **Length**  **(nt)** | **Reference** | **Read count** | | | |
| --- | --- | --- | --- | --- | --- | --- | --- | --- |
|  |  |  |  |  | **mock** | **drought** | **salinity** | **alkalinity** |
| 156 | gma-MIR156d* | GCTCACTTCTCTTTCTGTCA | 20 | zma-miR156d* | 2 | 2 | 1 | 0 |
|  | gma-MIR156f | TGACAGAAGAGAGAGAGCAC | 20 | gma-miR156f | 264 | 703 | 515 | 629 |
| 157 | gma-MIR157b* | GCTCTCTAAGCTTCTGTCAT | 20 | aly-miR157b* | 1 | 0 | 0 | 0 |
| 160 | gma-MIR160a | TGCCTGGCTCCCTGAATGCCA | 21 | vvi-miR160a | 13 | 20 | 11 | 0 |
| 162 | gma-MIR162* | TGGAGGCAGCGGTTCATCGATC | 22 | csi-miR162* | 171 | 285 | 220 | 125 |
| 166 | gma-MIR166a | TCGGACCAGGCTTCATTCCCG | 21 | crt-miR166a | 20283 | 25828 | 28491 | 24906 |
|  | gma-MIR166b | TCGGACCAGGCTTCATTCCCT | 21 | crt-miR166b | 10738 | 18470 | 19597 | 15406 |
|  | gma-MIR166 | TCGGACCAGGCTTCATTCCCC | 21 | ctr-miR166 | 161949 | 263470 | 281243 | 218063 |
| 167 | gma-MIR167a | TGAAGCTGCCAGCATGATCTGA | 22 | ccl-miR167a | 148 | 171 | 197 | 118 |
|  | gma-MIR167d | TGAAGCTGCCAGCATGATCTG | 21 | ath-miR167d | 8932 | 12392 | 13449 | 10665 |
| 169 | gma-MIR169d | TGAGCCAAGGATGACTTGCCG | 21 | gma-miR169d | 236 | 773 | 438 | 525 |
|  | gma-MIR169g | AGCCAAGGATGACTTGCC | 18 | bna-miR169g | 7 | 0 | 0 | 7 |
| 171 | gma-MIR171b-5p | CGGCGTGATATTGGTACGGCTC | 22 | gma-miR171b-5p | 21 | 79 | 22 | 28 |
| 172 | gma-MIR172d | GGAATCTTGATGATGCTGCA | 20 | gma-miR172d | 1 | 6403 | 2 | 3275 |
| 393 | gma-MIR393a | TCCAAAGGGATCGCATTG | 18 | ath-miR393a | 27 | 38 | 10 | 21 |
|  | gma-MIR393b | TCCAAAGGGATCGCATTGATCT | 22 | osa-miR393b | 2 | 2 | 2 | 0 |
| 394 | gma-MIR394a | TTGGCATTCTGTCCACCTCC | 20 | vvi-miR394a | 32 | 67 | 4 | 0 |
| 395 | gma-MIR395a | CTGAAGTGTTTGGGGGAACTC | 21 | sly-miR395a | 24 | 71 | 31 | 143 |
| 396 | gma-MIR396d | AAGAAAGCTGTGGGAGAATATGGC | 24 | gma-miR396d | 6 | 10 | 4 | 7 |
|  | gma-MIR396e | TTCCACAGCTTTCTTGAA | 18 | gma-miR396e | 3444 | 10754 | 5941 | 6727 |
| 397 | gma-MIR397a | TCATTGAGTGCAGCGTTGATG | 21 | bna-miR397a | 221 | 267 | 251 | 431 |
| 408 | gma-MIR408 | TGCACTGCCTCTTCCCTGGC | 20 | smo-miR408 | 29 | 38 | 22 | 46 |
| 482 | gma-MIR482a-3p | TTCCCAATTCCGCCCATTCCTA | 22 | gma-miR482a-3p | 133 | 150 | 260 | 255 |
|  | gma-MIR482a-5p | AGAATTTGTGGGAATGGGCTGA | 22 | gma-miR482a-5p | 69 | 63 | 68 | 107 |
|  | gma-MIR482b | TATGGGGGGATTGGGAAGGA | 20 | gma-miR482b | 7083 | 19939 | 8262 | 15345 |
|  | gma-MIR482* | GGAATGGGCTGATTGGGAAGC | 21 | pvu-miR482* | 25774 | 49213 | 36494 | 51713 |
| 829 | gma-MIR829.1 | CATCATTTGGTATCAGAG | 18 | ath-miR829.1 | 7 | 16 | 14 | 10 |
| 1507 | gma-MIR1507a | TCTCATTCCATACATCGTCTGA | 22 | gma-miR1507a | 303260 | 936627 | 727116 | 557141 |
| 1508 | gma-MIR1508a | TAGAAAGGGAAATAGCAGTTG | 21 | gma-miR1508a | 9370 | 27656 | 16432 | 16331 |
| 1509 | gma-MIR1509a | TTAATCAAGGAAATCACGGTCG | 22 | gma-miR1509a | 114830 | 366557 | 189909 | 228513 |
| 1510 | gma-MIR1510a-3p | TGTTGTTTTACCTATTCCACC | 21 | gma-miR1510a-3p | 1175 | 2994 | 1755 | 1893 |
|  | gma-MIR1510a-5p | AGGGATAGGTAAAACAATGAC | 21 | gma-miR1510a-5p | 16704 | 72810 | 32560 | 39686 |
| 1512 | gma-MIR1512 | TAACTGAAAATTCTTAAAGTA | 21 | gma-miR1512 | 321 | 393 | 176 | 183 |
| 1515 | gma-MIR1515 | TCATTTTGCGTGCAATGATCTG | 22 | gma-miR1515 | 136 | 477 | 317 | 205 |
| 1517 | gma-MIR1517 | AGTCTTGGTCAATGTCGTTCGAAA | 24 | gma-miR1517 | 1 | 0 | 0 | 3 |
| 1520 | gma-MIR1520c | AATAAGAACGTGACACGTG | 19 | gma-miR1520c | 3 | 4 | 5 | 0 |
|  | gma-MIR1520d | TCAGAACATGACACGTGACAA | 21 | gma-miR1520d | 2 | 14 | 4 | 7 |
|  | gma-MIR1520e | CAATAAGAACGTGACATATGACAG | 24 | gma-miR1520e | 2 | 14 | 10 | 10 |
|  | gma-MIR1520f | CAATCAGAACATGACACATGACAA | 24 | gma-miR1520f | 23 | 57 | 52 | 39 |
|  | gma-MIR1520g | CAATCAGAACATGACACGTGACAA | 24 | gma-miR1520g | 42 | 79 | 86 | 53 |
|  | gma-MIR1520h | AACGTCCAATCAGAACGTGACATG | 24 | gma-miR1520h | 1 | 4 | 1 | 3 |
|  | gma-MIR1520i | AACGTGACACGTGACGGTCAACAT | 24 | gma-miR1520i | 1 | 6 | 2 | 3 |
|  | gma-MIR1520j | AAGAACGTGACACATGACAATCAA | 24 | gma-miR1520j | 5 | 10 | 7 | 0 |
|  | gma-MIR1520k | AATCAGAACATGACACATGACAGT | 24 | gma-miR1520k | 25 | 89 | 49 | 32 |
|  | gma-MIR1520l | AATCAGAACATGACACGTGATAGT | 24 | gma-miR1520l | 35 | 120 | 80 | 118 |
|  | gma-MIR1520m | AATCAGAACATGACATGTGACAAT | 24 | gma-miR1520m | 10 | 18 | 2 | 3 |
|  | gma-MIR1520n | TCAATCAGAACATGACACGTGACA | 24 | gma-miR1520n | 21 | 50 | 50 | 53 |
|  | gma-MIR1520o | TGTCACATTCTGATTGGACGA | 21 | gma-miR1520o | 1 | 4 | 4 | 0 |
|  | gma-MIR1520p | ATGTTGTTATTGGATGATGACGGT | 24 | gma-miR1520p | 3 | 6 | 5 | 7 |
|  | gma-MIR1520q | ATTGACCAATCAGAACATGAC | 21 | gma-miR1520q | 7 | 24 | 29 | 46 |
|  | gma-MIR1520r | GTCACATCCTGGTTGGACATGAA | 23 | gma-miR1520r | 2 | 2 | 1 | 0 |
| 1526 | gma-MIR1526 | CGGAAGAGGAAAATTAAGCAA | 21 | gma-miR1526 | 1 | 0 | 0 | 0 |
| 1531 | gma-MIR1531 | TCGTCCATATGGGAAGACTTG | 21 | gma-miR1531 | 1 | 2 | 0 | 0 |
| 1863 | gma-MIR1863 | AATCTAACATGGTATCAGAGC | 21 | osa-miR1863 | 4 | 6 | 4 | 0 |
| 2089 | gma-MIR2089 | TACCTATTCCACCAATTCCAT | 21 | mtr-miR2089 | 5 | 6 | 2 | 3 |
| 2118 | gma-MIR2118 | TTGCCGATTCCACCCATTCCTA | 22 | pvu-miR2118 | 3556 | 5617 | 8397 | 10086 |
| 3522 | gma-MIR3522b | TGAGACCAAATGAGCAGCTGA | 21 | gso-miR3522b | 488034 | 538040 | 417117 | 683920 |
| 3630 | gma-MIR3630* | TGGGAATCTCTCTGATGC | 18 | vvi-miR3630* | 3 | 0 | 0 | 3 |
| 4340 | gma-MIR4340 | TGCAGAGATAGGGACGCGCT | 20 | gma-miR4340 | 4 | 4 | 1 | 0 |
| 4341 | gma-MIR4341 | TGTGTTGAAAGTTTAACATGACGG | 24 | gma-miR4341 | 1 | 14 | 7 | 0 |
| 4342 | gma-MIR4342 | ATCGACTTAGAATGTAGGA | 19 | gma-miR4342 | 2 | 16 | 10 | 17 |
| 4343 | gma-MIR4343a | AAAAAACTTACGGATCAAGTTGAT | 24 | gma-miR4343a | 1 | 0 | 0 | 0 |
|  | gma-MIR4343b | TTACAGATCAAGTTGATTCGGA | 22 | gma-miR4343b | 1 | 8 | 0 | 0 |
| 4344 | gma-MIR4344 | AAGTAGACATTCTAAGACGTTGCT | 24 | gma-miR4344 | 8 | 16 | 25 | 17 |
| 4345 | gma-MIR4345 | TAAGACGGAACTTACAAAGATT | 22 | gma-miR4345 | 268 | 783 | 539 | 475 |
| 4347 | gma-MIR4347 | AAGCTTCTTACGGATCAAGTTGAT | 24 | gma-miR4347 | 1 | 2 | 8 | 3 |
| 4349 | gma-MIR4349 | TATTGGCTAGAGATAAGACAAAGA | 24 | gma-miR4349 | 5 | 18 | 16 | 43 |
| 4351 | gma-MIR4351 | TTGGGATTCAGTTGGAGTTGG | 21 | gma-miR4351 | 44 | 99 | 50 | 129 |
| 4352 | gma-MIR4352a | ATTTCTAGGACATACTACGACGGT | 24 | gma-miR4352a | 4 | 10 | 4 | 0 |
|  | gma-MIR4352b | TAAAATGTAGACATTCTAAGACGG | 24 | gma-miR4352b | 11 | 36 | 20 | 21 |
| 4353 | gma-MIR4353 | CAAGTCGTAGCCGGTGTTATTACT | 24 | gma-miR4353 | 1 | 8 | 4 | 3 |
| 4357 | gma-MIR4357 | CAGTCGTGTGATTGTACGGTTCAT | 24 | gma-miR4357 | 1 | 6 | 4 | 3 |
| 4358 | gma-MIR4358 | CAGTGCATGACTATATCGCCAG | 22 | gma-miR4358 | 3 | 10 | 5 | 3 |
| 4359 | gma-MIR4359a | AACGAAGTGACTCTAACATCGGTT | 24 | gma-miR4359a | 2 | 6 | 0 | 0 |
|  | gma-MIR4359b | AACGCGTGATATGTTAACATCGGT | 24 | gma-miR4359b | 3 | 18 | 16 | 7 |
| 4360 | gma-MIR4360 | AGTTGACGTACGTACGGATTG | 21 | gma-miR4360 | 2 | 10 | 4 | 3 |
| 4361 | gma-MIR4361 | CCGGAAGAGACTTACGGATCAACT | 24 | gma-miR4361 | 6 | 20 | 43 | 3 |
| 4362 | gma-MIR4362 | CCTTAGGACAGACGTCATG | 19 | gma-miR4362 | 56 | 228 | 113 | 97 |
| 4363 | gma-MIR4363 | CGATTACCAGAAGGCTTATTAG | 22 | gma-miR4363 | 4 | 6 | 4 | 10 |
| 4364 | gma-MIR4364a | CGCGAGATCGCACGGAAGAAGGTT | 24 | gma-miR4364a | 19 | 50 | 34 | 35 |
|  | gma-MIR4364b | TAACAACAGCGGAAGAACCTTCTT | 24 | gma-miR4364b | 9 | 10 | 25 | 10 |
| 4365 | gma-MIR4365 | AAGAACTTCTTCCGCGAGATCGCA | 24 | gma-miR4365 | 7 | 16 | 7 | 14 |
| 4366 | gma-MIR4366 | CTACTTAGTAGAGATTTGTTGG | 22 | gma-miR4366 | 14 | 50 | 32 | 57 |
| 4367 | gma-MIR4367 | CTGAACCCTAGCGAAGTAAATC | 22 | gma-miR4367 | 16 | 44 | 16 | 10 |
| 4368 | gma-MIR4368b | AAGGACGGTACTTACGTAAGCAAC | 24 | gma-miR4368b | 6 | 0 | 1 | 3 |
| 4369 | gma-MIR4369 | GGATCAAGCTGATCCGGAAGTGGA | 24 | gma-miR4369 | 21 | 48 | 49 | 43 |
| 4370 | gma-MIR4370 | AGTAGACTCGTCCGATTTTGCGTA | 24 | gma-miR4370 | 10 | 8 | 10 | 10 |
| 4371 | gma-MIR4371a | AAGTGATGACATGACAAGCGAAGT | 24 | gma-miR4371a | 10 | 2 | 1 | 10 |
|  | gma-MIR4371b | AAGTGATGACGTGGTAGACGGAGT | 24 | gma-miR4371b | 10 | 26 | 22 | 7 |
|  | gma-MIR4371c | GACGTGACAGACGGAATATCACAT | 24 | gma-miR4371c | 1 | 10 | 8 | 7 |
| 4373 | gma-MIR4373 | AAGTTGACGTACGTACGGATTGAC | 24 | gma-miR4373 | 1 | 8 | 0 | 3 |
| 4374 | gma-MIR4374a | TAAGACGGTCGTGATGTCAGCA | 22 | gma-miR4374a | 2 | 0 | 4 | 0 |
|  | gma-MIR4374b | TCAACAACGTCTTTGAAAG | 19 | gma-miR4374b | 7 | 36 | 41 | 46 |
| 4375 | gma-MIR4375 | TACCACTAGTGGTCGCGCCTGGCA | 24 | gma-miR4375 | 3 | 12 | 2 | 0 |
| 4376 | gma-MIR4376 | ACGCAGGAGAGATGACGCTGT | 21 | gma-miR4376 | 5 | 6 | 0 | 3 |
| 4377 | gma-MIR4377 | TACGTCATCGCTGAATGGAAGACG | 24 | gma-miR4377 | 1 | 2 | 2 | 3 |
| 4378 | gma-MIR4378a | ATAGGACTGTCTTAGAATGGTGTA | 24 | gma-miR4378a | 3 | 16 | 8 | 21 |
|  | gma-MIR4378b | TAGAACTGTCTTAGAATGTGCTAC | 24 | gma-miR4378b | 5 | 10 | 8 | 7 |
| 4379 | gma-MIR4379 | TAGAGTGTATACTGTGAGAGGCCT | 24 | gma-miR4379 | 5 | 18 | 11 | 10 |
| 4380 | gma-MIR4380a | CGGATTGTTGATCCGTATGTGCAT | 24 | gma-miR4380a | 1 | 12 | 10 | 3 |
|  | gma-MIR4380b | TCAACAATCCGTATGACCATA | 21 | gma-miR4380b | 1 | 2 | 0 | 0 |
| 4381 | gma-MIR4381 | TATGTGACGGTAAACGGTGACAAG | 24 | gma-miR4381 | 1 | 2 | 2 | 0 |
| 4382 | gma-MIR4382 | TATGTTAACTGATTTCATGGAT | 22 | gma-miR4382 | 1 | 2 | 2 | 0 |
| 4383 | gma-MIR4383 | GACCGGTTCAACTGAGATCCAATA | 24 | gma-miR4383 | 1 | 2 | 5 | 0 |
| 4384 | gma-MIR4384 | AATCAGACACTGCATTCAAAGACG | 24 | gma-miR4384 | 32 | 53 | 61 | 53 |
| 4385 | gma-MIR4385 | AATCGATGTAGAAAAGTGATTGGT | 24 | gma-miR4385 | 20 | 65 | 49 | 57 |
| 4386 | gma-MIR4386 | TCGAAGGTTCTGGAGAGGACTGCA | 24 | gma-miR4386 | 1 | 0 | 1 | 0 |
| 4387 | gma-MIR4387a | AACAAGACGTGATGACGTGACACT | 24 | gma-miR4387a | 5 | 16 | 13 | 0 |
|  | gma-MIR4387b | AAGGTGTGATGGCATGACACTCTG | 24 | gma-miR4387b | 7 | 42 | 31 | 39 |
|  | gma-MIR4387c | AGCGTGATGACGTGACACTC | 20 | gma-miR4387c | 3 | 4 | 5 | 3 |
| 4388 | gma-MIR4388 | AATCTTAGGGACCAAATTGACAGC | 24 | gma-miR4388 | 2 | 2 | 0 | 0 |
| 4390 | gma-MIR4390 | GTACTCGTCGGGTATCGGGTAT | 22 | gma-miR4390 | 5 | 22 | 5 | 3 |
| 4391 | gma-MIR4391 | TCTCGGCAAAGAACTAAGAAGA | 22 | gma-miR4391 | 5 | 22 | 11 | 7 |
| 4393 | gma-MIR4393a | TGAGAAAAGGACGGCAGAAAAGCC | 24 | gma-miR4393a | 10 | 20 | 20 | 10 |
|  | gma-MIR4393b | TTGAAAAGGGACAGCAGAGAAGCC | 24 | gma-miR4393b | 15 | 48 | 28 | 28 |
| 4394 | gma-MIR4394 | AATGGACTAAAGAGAAAGGGGCCG | 24 | gma-miR4394 | 1 | 6 | 2 | 10 |
| 4395 | gma-MIR4395 | TGGATAGGAGTATGGGCTTGAG | 22 | gma-miR4395 | 16 | 28 | 10 | 17 |
| 4396 | gma-MIR4396 | TGTAGTTTCTAAGACGATGCTGAC | 24 | gma-miR4396 | 5 | 24 | 19 | 3 |
| 4397 | gma-MIR4397 | TGTCAAAGATGTGGCGAATAC | 21 | gma-miR4397 | 84 | 261 | 281 | 212 |
| 4398 | gma-MIR4398 | TGTCAGCGGAGTGAGAAGACGAAA | 24 | gma-miR4398 | 8 | 44 | 22 | 7 |
| 4399 | gma-MIR4399 | TAACGAAAAAGGACTAACGAC | 21 | gma-miR4399 | 4 | 10 | 8 | 0 |
| 4400 | gma-MIR4400 | TTCGGAAAAATTCTGGAAGACGTC | 24 | gma-miR4400 | 4 | 16 | 7 | 7 |
| 4401 | gma-MIR4401 | ACAACGTCTTTGAAAGTAGGCATT | 24 | gma-miR4401 | 1 | 12 | 13 | 10 |
| 4402 | gma-MIR4402 | CATATTATGGGTCTCAGACGGA | 22 | gma-miR4402 | 1 | 0 | 1 | 0 |
| 4403 | gma-MIR4403 | ACGGACACCGAACACGACACGGAC | 24 | gma-miR4403 | 1 | 2 | 4 | 0 |
| 4404 | gma-MIR4404 | TCGTGGAAGACTGGCGGATCAA | 22 | gma-miR4404 | 1 | 4 | 10 | 3 |
| 4405 | gma-MIR4405 | ATTCTAAGACGGTTATCTGGGACC | 24 | gma-miR4405 | 2 | 20 | 13 | 10 |
| 4406 | gma-MIR4406 | ATTGATTCTGAGAGAACCGGTGTA | 24 | gma-miR4406 | 1 | 10 | 8 | 3 |
| 4407 | gma-MIR4407 | CAGAGGAAGCAGCACTTGTACC | 22 | gma-miR4407 | 6 | 22 | 26 | 28 |
| 4408 | gma-MIR4408 | TAACAACATTGGATGAGGGTTGGA | 24 | gma-miR4408 | 9 | 38 | 16 | 43 |
| 4409 | gma-MIR4409 | AACAAGTGGGTTTGTTGACTG | 21 | gma-miR4409 | 95 | 308 | 161 | 241 |
| 4410 | gma-MIR4410 | TATGTTGATCCGTATGAGTCGTAC | 24 | gma-miR4410 | 3 | 10 | 4 | 3 |
| 4411 | gma-MIR4411 | TTATTGTAACTAATTTGT | 18 | gma-miR4411 | 1 | 4 | 7 | 10 |
