# Supplementary material for: Characterization of the stress associated microRNAs in Glycine max by deep sequencing
Source: BMC Plant Biol. 2011 Nov 23;11:170. doi: 10.1186/1471-2229-11-170 (PMC3267681; doi:10.1186/1471-2229-11-170)
Supplement: Additional file 5 — Distribution of expressed miRNA tags. Number of expressed miRNA tags distributed in mock and three stresses, drought, salinity, and alkalinity. [file 1471-2229-11-170-S5.DOC]

Additional file 5:

| **Name** | **mock** | | **drought** | | **salinity** | | **alkalinity** | |
| --- | --- | --- | --- | --- | --- | --- | --- | --- |
| gma-MIR482b | 7083 | 0.6016% | 19939 | 0.8442% | 8262 | 0.4614% | 15345 | 0.8129% |
| gma-MIR482a-5p | 69 | 0.0059% | 63 | 0.0027% | 68 | 0.0038% | 107 | 0.0057% |
| gma-MIR482a-3p | 133 | 0.0113% | 150 | 0.0064% | 260 | 0.0145% | 255 | 0.0135% |
| gma-MIR482* | 25774 | 2.189% | 49213 | 2.0836% | 36494 | 2.0382% | 51713 | 2.7395% |
| gma-MIR4409 | 95 | 0.0081% | 308 | 0.013% | 161 | 0.009% | 241 | 0.0128% |
| gma-MIR4397 | 84 | 0.0071% | 261 | 0.0111% | 281 | 0.0157% | 212 | 0.0112% |
| gma-MIR4362 | 56 | 0.0048% | 228 | 0.0097% | 113 | 0.0063% | 97 | 0.0051% |
| gma-MIR4351 | 44 | 0.0037% | 99 | 0.0042% | 50 | 0.0028% | 129 | 0.0068% |
| gma-MIR4345 | 268 | 0.0228% | 783 | 0.0332% | 539 | 0.0301% | 475 | 0.0252% |
| gma-MIR397a | 221 | 0.0188% | 267 | 0.0113% | 251 | 0.014% | 431 | 0.0228% |
| gma-MIR396e | 3444 | 0.2925% | 10754 | 0.4553% | 5941 | 0.3318% | 6727 | 0.3564% |
| gma-MIR395a | 24 | 0.002% | 71 | 0.003% | 31 | 0.0017% | 143 | 0.0076% |
| gma-MIR3522b | 488034 | 41.4488% | 538040 | 22.7798% | 417117 | 23.2966% | 683920 | 36.231% |
| gma-MIR2118 | 3556 | 0.302% | 5617 | 0.2378% | 8397 | 0.469% | 10086 | 0.5343% |
| gma-MIR172d | 1 | 0.0001% | 6403 | 0.2711% | 2 | 1e-04% | 3275 | 0.1735% |
| gma-MIR169d | 236 | 0.02% | 773 | 0.0327% | 438 | 0.0245% | 525 | 0.0278% |
| gma-MIR167d | 8932 | 0.7586% | 12392 | 0.5247% | 13449 | 0.7511% | 10665 | 0.565% |
| gma-MIR167a | 148 | 0.0126% | 171 | 0.0072% | 197 | 0.011% | 118 | 0.0063% |
| gma-MIR166b | 10738 | 0.912% | 18470 | 0.782% | 19597 | 1.0945% | 15406 | 0.8161% |
| gma-MIR166a | 20283 | 1.7226% | 25828 | 1.0935% | 28491 | 1.5913% | 24906 | 1.3194% |
| gma-MIR166 | 161949 | 13.7544% | 263470 | 11.1549% | 281243 | 15.7078% | 218063 | 11.552% |
| gma-MIR162* | 171 | 0.0145% | 285 | 0.0121% | 220 | 0.0123% | 125 | 0.0066% |
| gma-MIR156f | 264 | 0.0224% | 703 | 0.0298% | 515 | 0.0288% | 629 | 0.0333% |
| gma-MIR1520l | 35 | 0.003% | 120 | 0.0051% | 80 | 0.0045% | 118 | 0.0063% |
| gma-MIR1515 | 136 | 0.0116% | 477 | 0.0202% | 317 | 0.0177% | 205 | 0.0109% |
| gma-MIR1512 | 321 | 0.0273% | 393 | 0.0166% | 176 | 0.0098% | 183 | 0.0097% |
| gma-MIR1510a-5p | 16704 | 1.4187% | 72810 | 3.0827% | 32560 | 1.8185% | 39686 | 2.1024% |
| gma-MIR1510a-3p | 1175 | 0.0998% | 2994 | 0.1268% | 1755 | 0.098% | 1893 | 0.1003% |
| gma-MIR1509a | 114830 | 9.7525% | 366557 | 15.5194% | 189909 | 10.6067% | 228513 | 12.1056% |
| gma-MIR1508a | 9370 | 0.7958% | 27656 | 1.1709% | 16432 | 0.9178% | 16331 | 0.8651% |
| gma-MIR1507a | 303260 | 25.7559% | 936627 | 39.6553% | 727116 | 40.6105% | 557141 | 29.5149% |
