# Supplementary material for: Characterization of the stress associated microRNAs in Glycine max by deep sequencing
Source: BMC Plant Biol. 2011 Nov 23;11:170. doi: 10.1186/1471-2229-11-170 (PMC3267681; doi:10.1186/1471-2229-11-170)
Supplement: Additional file 7 — Prediction of novel miRNAs. Novel miRNAs identified from soybean. [file 1471-2229-11-170-S7.DOC]

Additional file 7. The novel miRNAs predicted from both arms of the miRNA precursor

| **miRID** | **Location** | **Strand**  **(+/-)** | **Energy**  (kcal/mol) | **Sequence of 5p** | **Sequence of 3p** | **Mock (count)** | | **Drought (count)** | | **Salinity (count)** | | **Alkalinity (count)** | |
| --- | --- | --- | --- | --- | --- | --- | --- | --- | --- | --- | --- | --- | --- |
|  |  |  |  |  |  | **5p** | **3p** | **5p** | **3p** | **5p** | **3p** | **5p** | **3p** |
| Gma-m001 | Gm18:61442586:61442692 | - | -44.1 | CTGACAGAAGATAGAGAGCAC | - | 3395 | - | 3248 | - | 4830 | - | 2216 |  |
| Gma-m002 | Gm02:837420:837549 | + | -56.5 | CAGGGGAACAGGCAGAGCATG | - | 3672 | - | 2573 | - | 2945 | - | 3159 |  |
| Gma-m003 | Gm12:3176108:3176377 | + | -69.67 | TCCATTGTCGTCCAGCGGTTA | - | 3282 | - | 3673 | - | 2931 | - | 1186 |  |
| Gma-m004 | Gm19:40699070:40699221 | - | -65.8 | TGGGTGAGAGAAACGCGTATC | TACGGGTCGCTCTCACCTAGG | 367 | 879 | 491 | 1053 | 186 | 1083 | 125 |  |
| Gma-m005 | Gm14:5324794:5324912 | + | -44 | AGCCAAGAATGACTTGCCGGAA | CGGGCAAGTTGTTTTTGGCTAC | 337 | 560 | 475 | 644 | 438 | 471 | 175 |  |
| Gma-m006 | Gm09:16565920:16566038 | - | -44.7 | AGAGGTGTTTGGGATGAGAGA | CCTCATTCCAAACATCATCTAA | 1596 | 102 | 1695 | 138 | 777 | 128 | 335 |  |
| Gma-m007 | Gm18:61452908:61452997 | - | -41 | GGAATGGGCTGATTGGGAAGT | - | 835 | - | 781 | - | 813 | - | 598 |  |
| Gma-m008 | Gm02:30498945:30499130 | - | -70.5 | CTGGGTGAGAGAAACACGTAT | ACGGGTCGCTCTCACCTGGAG | 85 | 665 | 170 | 635 | 78 | 714 | 43 |  |
| Gma-m009 | Gm13:34382988:34383131 | - | -57.37 | TCATTGAGTGCAGCGTTGATG | TATTGACGCTGCACTCAATCA | 332 | 811 | 187 | 744 | 202 | 340 | 142 |  |
| Gma-m010 | Gm06:10859290:10859391 | - | -33.76 | - | CGAGCCGAATCAATACCACTC | - | 658 | - | 693 | - | 515 | - |  |
| Gma-m011 | Gm17:9101686:9101811 | - | -48.4 | CGATGTTGGTGAGGTTCAATC | TTGAGCCGCGCCAATATCACT | 3 | 633 | - | 659 | 1 | 506 | 19 |  |
| Gma-m012 | Gm03:25186869:25186990 | + | -59.9 | TCACGCCTAATCACTGACGCAT | TGTTAGTGATAAGGCGTGATG | - | 534 | - | 673 | - | 418 | 126 | 25 |
| Gma-m013 | Gm08:10105804:10105875 | + | -24.2 | CGTGACTTGAACAGCTACGTA | - | 274 | - | 497 | - | 347 | - | 102 | - |
| Gma-m014 | Gm18:61624595:61624704 | - | -42.5 | TCCCTCAAAGGCTTCCAGTAT | GCTGGATGTCTTTGAAGGAAT | - | 162 | - | 173 | - | 322 | 47 | 38 |
| Gma-m015 | Gm07:10001915:10001997 | + | -41.1 | TTATCAGTAGCATCATCATCA | - | 199 | - | 402 | - | 323 | - | 75 | - |
| Gma-m016 | Gm05:41564645:41564756 | + | -42.4 | TGTTGGACGGTTCAATCAAA | TGATTGAGCCGTGCCAATATC | 251 | 111 | 339 | 148 | 203 | 108 | 38 | 11 |
| Gma-m017 | Gm18:61655979:61656073 | - | -49.2 | CCTCAAAGGCTTCCACTACTG | GTAGTGGATGCCTAGAGGTC | - | 154 | - | 166 | - | 309 | 10 | 21 |
| Gma-m018 | Gm10:2905320:2905422 | - | -57.6 | GGAATGTCGTTTGGTTCGAGA | - | 98 | 195 | 98 | 214 | 98 | 212 | 283 | - |
| Gma-m019 | Gm11:32789075:32789193 | - | -54.5 | CATGGAAGTGAATCGGGTGAC | - | - | 277 | - | 172 | - | 280 | 234 | - |
| Gma-m020 | Gm09:109565:109722 | - | -53.3 | - | TTATGGAAGAAGCTCAATTCA | 259 | 12 | 334 | 7 | 246 | 7 | - | 41 |
| Gma-m021 | Gm07:10004527:10004609 | + | -40.6 | TTATCAGTAGCATCATCATCA | - | 259 | 11 | 335 | 3 | 246 | 8 | 75 | - |
| Gma-m022 | Gm07:35780689:35780937 | + | -52.2 | - | TCATTGAGTGTAGCATTGATG | 259 | 12 | 333 | 14 | 246 | 8 | - | 42 |
| Gma-m023 | Gm17:41783752:41783897 | + | -68.2 | - | AGCTCTGTTGGCTACACTTT | 246 | 1 | 314 | 1 | 233 | - | - | 156 |
| Gma-m024 | Gm13:20521462:20521566 | + | -50.8 | TGACAGAAGAGAGTGAGCACT | - | 331 | 5 | 185 | 3 | 202 | 5 | 82 | - |
| Gma-m025 | Gm01:31787443:31787550 | + | -50.7 | TAATCTGCATCCTGAGGTTTA | GTCCTTGGGATGCAGATTACG | - | 248 | 1 | 273 | 1 | 201 | 71 | 117 |
| Gma-m026 | Gm15:14171023:14171102 | + | -39.2 | TGAGCCAAGGATGACTTGCCG | - | 230 | - | 279 | 1 | 200 | 1 | 123 | - |
| Gma-m027 | Gm15:14191188:14191285 | + | -55.4 | TGAGCCAAGGATGACTTGCCG | GCGAGACATCTTGGCTCACT | 230 | - | 279 | - | 200 | - | 128 | 4 |
| Gma-m028 | Gm13:40315639:40315789 | - | -52.6 | TGCATTTGCACCTGCACTTTA | - | 230 | - | 279 | - | 200 | - | 108 | - |
| Gma-m029 | Gm06:37942221:37942403 | - | -61.7 | - | AAAAGCACTTAAGGAACGGTA | 367 | - | 491 | - | 186 | - | - | 98 |
| Gma-m030 | Gm13:40319194:40319358 | - | -71.3 | TGCATTTGCACCTGCACTTTA | - | 92 | 107 | 78 | 146 | 102 | 71 | 108 | - |
| Gma-m031 | Gm04:46988892:46988993 | + | -38.1 | - | CGAGCCGAATCAATACCACTC | - | 207 | - | 337 | - | 174 | - | 343 |
| Gma-m032 | Gm03:38094628:38094771 | - | -59.7 | TGGGTGAGAGAAACGCGTATC | - | - | 207 | - | 337 | - | 174 | 125 | - |
| Gma-m033 | Gm09:37395782:37395989 | - | -39.8 | - | TAATTGTGTTGTACATTATCA | 444 | - | 172 | - | 167 | - | - | 92 |
| Gma-m034 | Gm16:28980105:28980217 | + | -25.4 | - | AGCAATGGAATTATAGACTGC | - | 197 | - | 295 | - | 161 | - | 93 |
| Gma-m035 | Gm09:5285243:5285356 | + | -54.8 | TGAGCCAAGGATGACTTGCCG | GCGAGACATCTTGGTTCATT | - | 259 | - | 55 | - | 137 | 128 | 7 |
| Gma-m036 | Gm07:16377794:16377881 | - | -37.6 | TAATCTGCATCCTGAGGTTTA | - | - | 154 | - | 153 | - | 135 | 71 | - |
| Gma-m037 | Gm02:14614766:14614989 | - | -54.8 | - | TTTGGTCTTTAATCAAGCTGA | 105 | - | 174 | - | 130 | 1 | - | 45 |
| Gma-m038 | Gm14:13819007:13819089 | + | -42.7 | TTATCAGTAGCATCATCATCA | - | 481 | 43 | 188 | 38 | 120 | 12 | 75 | - |
| Gma-m039 | Gm04:25372869:25373018 | + | -49 | - | TCGGACCAGGCTTCATTCCCCA | 106 | - | 175 | - | 129 | 1 | - | 117 |
| Gma-m040 | Gm02:44954758:44954854 | + | -40 | AAGCTCAGGAGGGATAGCACCA | - | 106 | - | 175 | - | 129 | 1 | 29 | - |
| Gma-m041 | Gm09:5282116:5282208 | + | -51.2 | TGAGCCAAGGATGACTTGCCG | GCGAGACATCTTGGCTCATT | 106 | - | 175 | - | 129 | - | 128 | 4 |
| Gma-m042 | Gm16:28931061:28931173 | + | -24.7 | - | AGCAATGGAATTATAGACTGC | 47 | - | 122 | - | 104 | - | - | 89 |
| Gmam0043 | Gm18:49934393:49934476 | - | -44.6 | TAATCTGCATCCTGAGGTTTA | - | 97 | - | 98 | - | 98 | - | 71 | - |
| Gmam0044 | Gm13:35514890:35515064 | + | -94.1 | - | TCAATCCTGGAAGAACCGGCG | 97 | - | 98 | - | 98 | - | - | 90 |
| Gmam0045 | Gm13:26271122:26271247 | + | -46.3 | CGATGTTGGTGAGGTTCAATC | TTGAGCCGCGCCAATATCACT | 58 | 65 | 55 | 40 | 42 | 51 | 19 | 17 |
| Gmam0046 | Gm12:33689553:33689722 | + | -64.3 | TGCATTTGCACCTGCACTTTA | - | 58 | 65 | 55 | 40 | 42 | 51 | 108 | - |
| Gmam0047 | Gm13:363845:363943 | - | -43.9 | TGAGCCAGGATGGCTTGCCGGC | TGGTGGCCATCCATGCTCATA | 99 | 2 | 130 | 1 | 74 | - | 44 | 2 |
| Gmam0048 | Gm08:4639045:4639154 | - | -46.4 | TCATTGAGTGCAGCGTTGATG | TCGACACTGCACTCAATCATG | - | 78 | - | 121 | - | 69 | 142 | 3 |
| Gmam0049 | Gm20:32872633:32872816 | + | -66.5 | - | AAAAGCACTTAAGGAACGGTA | 54 | - | 61 | - | 52 | - | - | 98 |
| Gmam0050 | Gm11:16833248:16833431 | - | -70.8 | TGCATTTGCACCTGCACTTTA | - | 47 | 57 | 41 | 112 | 26 | 16 | 109 | - |
