# Supplementary material for: Characterization of the stress associated microRNAs in Glycine max by deep sequencing
Source: BMC Plant Biol. 2011 Nov 23;11:170. doi: 10.1186/1471-2229-11-170 (PMC3267681; doi:10.1186/1471-2229-11-170)
Supplement: Additional file 8 — The predicted miRNA targeted genes in Glycine max. [file 1471-2229-11-170-S8.DOC]

**Additional file 8:** **The predicted miRNA targeted genes in *Glycine max***

| miRNA ID | Targeted genes | Functional description |
| --- | --- | --- |
| gma-miR156d | FK553291 | transcriptional regulator |
| gma-miR156d | TC357522 | Peptidyl-prolyl cis-trans isomerase |
| gma-miR156d | BM523406 | Hydroxyproline-rich glycoprotein |
| gma-miR156d | TC389916 | Quinone oxidoreductase-like protein |
| gma-miR156d | TC371676 | Hypothesis protein |
| gma-miR156d | GD762235 | Hypothesis protein |
| gma-miR156d | EH221204 | Hypothesis protein |
| gma-miR156d | AW831126 | Hypothesis protein |
| gma-miR156d | TC370573 | Bifunctional dihydrofolate reductase-thymidylate synthase (DHFR-TS) |
| gma-miR156d | TC405780 | Predicted protein |
| gma-miR156d | TC360158 | PRLI-interacting factor A |
| gma-miR156d | GE016857 | Predicted protein |
| gma-miR156d | GD759449 | Predicted protein |
| gma-miR156d | GE097462 | Predicted protein |
| gma-miR156d | TC413002 | Predicted protein |
| gma-miR156d | FK628156 | Predicted protein |
| gma-miR156f | TC393941 | Predicted protein |
| gma-miR156f | TC353286 | Predicted protein |
| gma-miR156f | TC407577 | SBP transcription factor |
| gma-miR156f | TC378777 | Predicted protein |
| gma-miR156f | TC393578 | Squamosa promoter-binding-like protein 6 |
| gma-miR156f | TC373253 | Squamosa promoter-binding-like protein 13 |
| gma-miR156f | TC353528 | Olfactory receptor Olfr645 |
| gma-miR156f | TC414152 | Predicted protein |
| gma-miR156f | TC407338 | Hypothesis protein |
| gma-miR156f | BE329522 | Biotoin carboxyl carrier protein |
| gma-miR156f | TC371785 | Conserved protein with cbs domain |
| gma-miR156f | TC419101 | Hypothesis protein |
| gma-miR156f | FK569365 | Hypothesis protein |
| gma-miR156f | AW307452 | Squamosa promoter binding-like protein |
| gma-miR156f | TC406460 | Hypothesis protein |
| gma-miR156f | TC414827 | Hypothesis protein |
| gma-miR156f | TC393424 | SBP transcription factor |
| gma-miR156f | TC398968 | Squamosa promoter-binding-like protein 1 |
| gma-miR156f | TC358816 | AT-hook DNA-binding protein |
| gma-miR156f | DB962750 | AT-hook DNA-binding protein |
| gma-miR156f | TC392816 | Hypothesis protein |
| gma-miR156f | TC386226 | SBP transcription factor |
| gma-miR156f | EV277456 | Hypothesis protein |
| gma-miR156f | TC370293 | Hypothesis protein |
| gma-miR157b | CD393563 | Hypothesis protein |
| gma-miR157b | TC405317 | Hypothesis protein |
| gma-miR157b | TC397626 | Hypothesis protein |
| gma-miR157b | TC376154 | RecA bacterial DNA recombination protein |
| gma-miR157b | TC381658 | Hypothesis protein |
| gma-miR157b | GE027615 | Hypothesis protein |
| gma-miR157b | TC419404 | Protein kinase-like protein |
| gma-miR157b | TC413563 | Hypothesis protein |
| gma-miR157b | TC373269 | Hypothesis protein |
| gma-miR157b | BU081426 | Pedal peptide 4 |
| gma-miR157b | NP8367007 | polyphenol oxidase |
| gma-miR157b | TC351733 | Hypothesis protein |
| gma-miR157b | TC393799 | Hypothesis protein |
| gma-miR157b | TC411489 | Hypothesis protein |
| gma-miR160a | TC358753 | Auxin response factor 17 |
| gma-miR160a | TC350243 | Hypothesis protein |
| gma-miR160a | TC374356 | Hypothesis protein |
| gma-miR160a | TC366343 | Hypothesis protein |
| gma-miR160a | BE346420 | K+ channel tetramerisation |
| gma-miR160a | TC353309 | Hypothesis protein |
| gma-miR162 | TC375931 | Hypothesis protein |
| gma-miR162 | TC418937 | Expressed protein |
| gma-miR162 | TC372945 | Expressed protein |
| gma-miR162 | TC366573 | Lectin-like receptor kinase 1 |
| gma-miR162 | TC400714 | Hypothesis protein |
| gma-miR162 | AW395856 | Ribulose bisphosphate carboxylase/oxygenase activase |
| gma-miR162 | TC363160 | Hypothesis protein |
| gma-miR162 | BU081804 | Membrane protein |
| gma-miR162 | TC360506 | Hypothesis protein |
| gma-miR162 | TC416891 | Hypothesis protein |
| gma-miR162 | GD897284 | Homeodomain-leucine zipper protein 56 |
| gma-miR162 | TC372993 | Homeodomain-leucine zipper protein 56 |
| gma-miR162 | FK550941 | Hypothesis protein |
| gma-miR166a | TC393445 | Hypothesis protein |
| gma-miR166a | TC400099 | Hypothesis protein |
| gma-miR166a | BM309730 | PHAVOLUTA-like HD-ZIPIII protein |
| gma-miR166a | TC396561 | Class III HD-Zip protein 4 |
| gma-miR166a | TC418837 | Hypothesis protein |
| gma-miR166a | TC409926 | CRK1 protein |
| gma-miR166a | TC407471 | Predicted protein |
| gma-miR166a | TC411802 | Hypothesis protein |
| gma-miR166a | TC385527 | Hypothesis protein |
| gma-miR166a | GD744384 | Type IIIa membrane protein cp-wap11 |
| gma-miR166a | TC355982 | Type IIIa membrane protein cp-wap11 |
| gma-miR166a | TC371581 | Type IIIa membrane protein cp-wap11 |
| gma-miR166a | TC352025 | Reversibly glycosylated protein |
| gma-miR166a | TC403858 | Reversibly glycosylated protein |
| gma-miR166a | TC348844 | Hypothesis protein |
| gma-miR166a | TC350487 | Hypothesis protein |
| gma-miR166a | TC418458 | Phosphoethanolamine N-methyltransferase |
| gma-miR166b | TC393445 | Hypothesis protein |
| gma-miR166b | BM309730 | PHAVOLUTA-like HD-ZIPIII protein |
| gma-miR166b | TC396561 | Class III HD-Zip protein 4 |
| gma-miR166b | TC400099 | Hypothesis protein |
| gma-miR166b | TC409926 | CRK1 protein |
| gma-miR166b | TC418837 | Hypothesis protein |
| gma-miR166b | TC407471 | Predicted protein |
| gma-miR166b | TC411802 | Hypothesis protein |
| gma-miR166b | TC385527 | Hypothesis protein |
| gma-miR166b | TC404342 | Isoamylase-type starch-debranching enzyme 1 |
| gma-miR166b | TC399552 | SET domain-containing protein-like |
| gma-miR166b | GD744384 | Type IIIa membrane protein cp-wap11 |
| gma-miR166b | TC355982 | Type IIIa membrane protein cp-wap11 |
| gma-miR166 | TC393445 | Hypothesis protein |
| gma-miR166 | BM309730 | PHAVOLUTA-like HD-ZIPIII protein |
| gma-miR166 | TC396561 | Class III HD-Zip protein 4 |
| gma-miR166 | TC400099 | Hypothesis protein |
| gma-miR166 | TC418837 | Hypothesis protein |
| gma-miR166 | TC409926 | CRK1 protein |
| gma-miR166 | TC404342 | Isoamylase-type starch-debranching enzyme 1 |
| gma-miR166 | TC399552 | SET domain-containing protein-like |
| gma-miR166 | TC407471 | Predicted protein |
| gma-miR166 | TC411802 | Hypothesis protein |
| gma-miR166 | TC385527 | Hypothesis protein |
| gma-miR167a | BE805600 | Auxin response factor 8 |
| gma-miR167a | TC371467 | Phosphatidate cytidylyltransferase |
| gma-miR167a | TC379788 | Hypothesis protein |
| gma-miR167a | TC371879 | Hypothesis protein |
| gma-miR167a | BM732289 | Hypothesis protein |
| gma-miR167a | TC389689 | Hypothesis protein |
| gma-miR167a | TC361615 | Hypothesis protein |
| gma-miR167a | TC412492 | Hypothesis protein |
| gma-miR167a | TC373430 | Hypothesis protein |
| gma-miR167a | TC384203 | Hypothesis protein |
| gma-miR167a | DB979348 | Hypothesis protein |
| gma-miR167a | TC365699 | Hypothesis protein |
| gma-miR167a | TC365019 | Hypothesis protein |
| gma-miR167a | TC391866 | Hypothesis protein |
| gma-miR167a | GD753695 | Hypothesis protein |
| gma-miR167d | TC371467 | Phosphatidate cytidylyltransferase |
| gma-miR167d | TC379788 | Hypothesis protein |
| gma-miR167d | TC371879 | Hypothesis protein |
| gma-miR167d | BE805600 | Auxin response factor 8 |
| gma-miR167d | BM732289 | Hypothesis protein |
| gma-miR167d | DB979348 | Hypothesis protein |
| gma-miR167d | TC389689 | Hypothesis protein |
| gma-miR167d | TC365699 | Hypothesis protein |
| gma-miR167d | TC361615 | Hypothesis protein |
| gma-miR167d | TC412492 | Hypothesis protein |
| gma-miR167d | TC373430 | Hypothesis protein |
| gma-miR167d | TC365019 | Hypothesis protein |
| gma-miR167d | TC384203 | Hypothesis protein |
| gma-miR167d | TC391866 | Hypothesis protein |
| gma-miR167d | GD753695 | Hypothesis protein |
| gma-miR169d | TC388709 | Hypothesis protein |
| gma-miR169d | TC358727 | YA3 |
| gma-miR169d | TC365692 | CCAAT-binding transcription factor |
| gma-miR169d | TC364843 | Hypothesis protein |
| gma-miR169d | TC353076 | Nuclear transcription factor Y subunit A-3 |
| gma-miR169d | TC379261 | Os02g0776400 protein |
| gma-miR169d | TC401273 | CCAAT-box transcription factor complex WHAP12 |
| gma-miR169d | TC355136 | Hypothesis protein |
| gma-miR169d | TC383014 | CCAAT-binding transcription factor |
| gma-miR169d | TC366077 | Hypothesis protein |
| gma-miR169d | CO985073 | Mitogen-activated protein kinase 10 |
| gma-miR171b-5p | AW349258 | Ubiquitin carrier protein |
| gma-miR171b-5p | AW760012 | Fe-superoxide dismutase 1 |
| gma-miR172d | FK382727 | Hypothesis protein |
| gma-miR172d | FK379774 | Hypothesis protein |
| gma-miR172d | TC405657 | Hypothesis protein |
| gma-miR172d | TC392019 | Transcription factor AHAP2 |
| gma-miR172d | BE659941 | Floral homeotic protein APETALA 2 |
| gma-miR172d | TC366837 | APETAL2-like protein |
| gma-miR172d | TC407080 | Transcription factor AHAP2 |
| gma-miR172d | TC378006 | Hypothesis protein |
| gma-miR172d | TC383306 | PHAP2B protein |
| gma-miR172d | TC404733 | Hypothesis protein |
| gma-miR172d | TC352579 | Hypothesis protein |
| gma-miR172d | TC383335 | PHAP2B protein |
| gma-miR172d | TC361830 | PHAP2B protein |
| gma-miR172d | TC349360 | Hypothesis protein |
| gma-miR172d | BI320499 | Hypothesis protein |
| gma-miR172d | BU084569 | Hypothesis protein |
| gma-miR172d | TC372191 | Hypothesis protein |
| gma-miR172d | FK339807 | APETAL2-like protein |
| gma-miR172d | TC395896 | Transcription factor AHAP2 |
| gma-miR172d | TC417910 | Superoxide dismutase [Cu-Zn] |
| gma-miR172d | TC405919 | Os04g0649100 protein |
| gma-miR172d | TC412825 | Transcription factor AHAP2 |
| gma-miR172d | TC412175 | APETAL2-like protein |
| gma-miR172d | CO980901 | Uncharacterized GTP-binding protein At5g64813 |
| gma-miR172d | GD721501 | Hypothesis protein |
| gma-miR393b | DB989850 | Auxin-responsive factor TIR1-like protein |
| gma-miR393b | TC416229 | Auxin-responsive factor TIR1-like protein |
| gma-miR393b | TC366828 | Transport inhibitor response 1 |
| gma-miR393b | TC365328 | Transport inhibitor response 1 |
| gma-miR393b | TC362546 | Transport inhibitor response 1 |
| gma-miR393b | TC398603 | Hypothesis protein |
| gma-miR393b | TC362758 | Hypothesis protein |
| gma-miR393b | FG999850 | Hypothesis protein |
| gma-miR393b | TC352212 | Hypothesis protein |
| gma-miR393b | TC350689 | Expressed protein |
| gma-miR393b | DB964070 | Hypothesis protein |
| gma-miR393b | TC370631 | Hypothesis protein |
| gma-miR393b | TC371702 | Hypothesis protein |
| gma-miR394a | TC400695 | Hypothesis protein |
| gma-miR394a | TC366476 | Hypothesis protein |
| gma-miR394a | TC388603 | Hypothesis protein |
| gma-miR394a | TC350576 | RNA-binding protein |
| gma-miR394a | TC352608 | Somatic embryogenesis receptor kinase |
| gma-miR394a | BE191081 | SLL1 protein |
| gma-miR394a | TC355756 | Hypothesis protein |
| gma-miR394a | TC373735 | MRNA, complete cds, clone: RAFL25-18-P16 |
| gma-miR394a | BM139543 | Actin depolymerizing factor-like protein |
| gma-miR395a | BG789910 | ATP sulfurylase |
| gma-miR395a | TC359920 | ATP sulfurylase |
| gma-miR395a | TC358067 | ATP sulfurylase |
| gma-miR395a | TC360687 | ATP sulfurylase |
| gma-miR395a | EV282501 | Hypothesis protein |
| gma-miR395a | GE008734 | Hypothesis protein |
| gma-miR395a | TC369301 | Hypothesis protein |
| gma-miR395a | TC349703 | Plastidial lipoyltransferase 2 |
| gma-miR395a | TC348882 | Plastidial lipoyltransferase 2 |
| gma-miR395a | TC357122 | Hypothesis protein |
| gma-miR395a | CF922366 | Phospholipase C |
| gma-miR395a | TC358694 | Low affinity sulfate transporter 3 |
| gma-miR395a | TC405478 | Hypothesis protein |
| gma-miR395a | TC411365 | Cation diffusion facilitator 9 |
| gma-miR395a | AW759383 | Glutamate dehydrogenase 2 |
| gma-miR395a | GR836780 | Hypothesis protein |
| gma-miR395a | TC369607 | Zinc finger, CCCH-type; Sugar transporter superfamily |
| gma-miR395a | TC357118 | Hypothesis protein |
| gma-miR395a | TC397169 | Zinc finger, CCCH-type; Sugar transporter superfamily |
| gma-miR395a | TC365139 | Ketol-acid reductoisomerase, chloroplast precursor |
| gma-miR395a | GD787823 | Dihydroflavonol-4-reductase DFR1 |
| gma-miR396d | TC365248 | Hypothesis protein |
| gma-miR396d | FK003100 | Hypothesis protein |
| gma-miR396d | TC373306 | Cytochrome P450 monooxygenase CYP72A65 |
| gma-miR396d | TC366659 | Hypothesis protein |
| gma-miR396d | TC393538 | Hypothesis protein |
| gma-miR396d | TC379767 | Hypothesis protein |
| gma-miR396d | TC393753 | Elovl2 protein |
| gma-miR396d | TC416103 | Hypothesis protein |
| gma-miR396d | TC374491 | Hypothesis protein |
| gma-miR396d | TC417292 | Hypothesis protein |
| gma-miR396d | FK473732 | NADH-ubiquinone oxidoreductase chain 4 |
| gma-miR396d | AW349891 | Hypothesis protein |
| gma-miR397a | TC378864 | Diphenol oxidase |
| gma-miR397a | TC354218 | Laccase-7 precursor |
| gma-miR397a | BE474231 | Hypothesis protein |
| gma-miR397a | TC414829 | Laccase-17 precursor |
| gma-miR397a | TC371812 | Hypothesis protein |
| gma-miR397a | TC348738 | Diphenol oxidase laccase |
| gma-miR397a | TC391633 | Diphenol oxidase |
| gma-miR397a | TC382844 | Laccase-7 precursor |
| gma-miR397a | TC381077 | Hypothesis protein |
| gma-miR397a | TC408946 | Hypothesis protein |
| gma-miR397a | FK018104 | Hypothesis protein |
| gma-miR397a | TC387507 | Hypothesis protein |
| gma-miR397a | TC351582 | Hypothesis protein |
| gma-miR397a | TC399860 | Acetyl-CoA synthetase |
| gma-miR397a | BU090696 | Hypothesis protein |
| gma-miR397a | TC391114 | Hypothesis protein |
| gma-miR397a | TC361643 | Hypothesis protein |
| gma-miR397a | TC392371 | Hypothesis protein |
| gma-miR397a | TC369680 | Hypothesis protein |
| gma-miR408 | TC413923 | Auxin down-regulated protein |
| gma-miR408 | TC385128 | Hypothesis protein |
| gma-miR408 | TC361540 | Basic blue copper protein |
| gma-miR408 | TC350484 | Basic blue copper protein |
| gma-miR408 | DB975351 | Hypothesis protein |
| gma-miR408 | DB985567 | Proteoglycan-4 precursor |
| gma-miR408 | BQ081204 | Basic blue copper protein |
| gma-miR408 | BI425573 | Basic blue copper protein |
| gma-miR408 | EV280522 | Laccase-3 precursor |
| gma-miR408 | TC419112 | kelch repeat-containing protein |
| gma-miR408 | TC419377 | Chloroplast stromal ascorbate peroxidase |
| gma-miR408 | FK403453 | Histone H2A |
| gma-miR408 | TC348738 | Diphenol oxidase laccase |
| gma-miR408 | TC363850 | Hypothesis protein |
| gma-miR408 | BQ080825 | Diphenol oxidase laccase |
| gma-miR482a-3p | TC404387 | Hypothesis protein |
| gma-miR482a-3p | DB967214 | Cytochrome c oxidase subunit 3 |
| gma-miR482a-3p | TC385797 | Hypothesis protein |
| gma-miR482a-3p | TC367553 | Hypothesis protein |
| gma-miR482a-3p | TC381004 | Glyoxal or galactose oxidase |
| gma-miR482a-3p | TC378949 | Phosphoribosylformylglycinamidine synthase subunit II |
| gma-miR482a-3p | TC417078 | Disease resistance-like protein |
| gma-miR482a-3p | EV262954 | NBS-LRR type disease resistance protein |
| gma-miR482a-3p | TC372980 | R 1 protein |
| gma-miR482a-3p | BI316773 | 50S ribosomal protein L3 |
| gma-miR482a-3p | TC374623 | Hypothesis protein |
| gma-miR482a-3p | GR843719 | Extensin |
| gma-miR482a-3p | TC420140 | 5,10-methylenetetrahydrofolate dehydrogenase-5,10- methenyltetrahydrofolate cyclohydrolase |
| gma-miR482a-3p | TC388919 | 5,10-methylenetetrahydrofolate dehydrogenase-5,10- methenyltetrahydrofolate cyclohydrolase |
| gma-miR482a-5p | BG507664 | Hypothesis protein |
| gma-miR482a-5p | TC363409 | Hypothesis protein |
| gma-miR482a-5p | TC375755 | Hypothesis protein |
| gma-miR482a-5p | TC367224 | Hypothesis protein |
| gma-miR482a-5p | TC367682 | Hypothesis protein |
| gma-miR482a-5p | TC385349 | Hypothesis protein |
| gma-miR482a-5p | TC404638 | Hypothesis protein |
| gma-miR482a-5p | TC420342 | Glucosyltransferase-3 |
| gma-miR482a-5p | TC408807 | Hypothesis protein |
| gma-miR482a-5p | TC394782 | Hypothesis protein |
| gma-miR482a-5p | TC357061 | Hypothesis protein |
| gma-miR482a-5p | TC351902 | Hypothesis protein |
| gma-miR482b | TC392949 | MADS box protein AGL11 |
| gma-miR482b | BU545647 | Hypothesis protein |
| gma-miR482b | TC420696 | Hypothesis protein |
| gma-miR482b | FK024491 | Hypothesis protein |
| gma-miR482b | FK296071 | Hypothesis protein |
| gma-miR482b | FK612472 | Hypothesis protein |
| gma-miR482b | FK470136 | Hypothesis protein |
| gma-miR482b | CF922521 | Hypothesis protein |
| gma-miR482b | TC417145 | Hypothesis protein |
| gma-miR482b | TC380393 | Hypothesis protein |
| gma-miR482b | TC376212 | Mitochondrial ribosomal protein S19 |
| gma-miR482b | FG989764 | Hypothesis protein |
| gma-miR482b | TC387186 | Hypothesis protein |
| gma-miR482b | TC370626 | Hypothesis protein |
| gma-miR482b | TC374018 | Hypothesis protein |
| gma-miR482b | TC355751 | CG15765-PA |
| gma-miR482b | TC360604 | Androgen receptor |
| gma-miR482b | TC380505 | Uncharacterized protein At2g35230.2 |
| gma-miR482b | TC351238 | Hypothesis protein |
| gma-miR482b | TC381275 | Hypothesis protein |
| gma-miR482b | TC365126 | Hypothesis protein |
| gma-miR482b | TC407392 | Hypothesis protein |
| gma-miR482b | TC361059 | Hypothesis protein |
| gma-miR482b | FK554786 | Hypothesis protein |
| gma-miR482b | GD739896 | Protein translation factor SUI1 homolog |
| gma-miR482 | TC380505 | Uncharacterized protein At2g35230.2 |
| gma-miR482 | BE474774 | Peroxisomal copper-containing amine oxidase |
| gma-miR482 | BU548045 | Peroxisomal copper-containing amine oxidase |
| gma-miR482 | TC377668 | Hypothesis protein |
| gma-miR482 | TC369735 | Hypothesis protein |
| gma-miR482 | TC352725 | Hypothesis protein |
| gma-miR482 | TC365299 | Hypothesis protein |
| gma-miR482 | TC357803 | Hypothesis protein |
| gma-miR482 | TC412104 | Hypothesis protein |
| gma-miR482 | TC367224 | Hypothesis protein |
| gma-miR482 | TC367682 | Hypothesis protein |
| gma-miR482 | FK623745 | Hypothesis protein |
| gma-miR482 | TC366590 | Hypothesis protein |
| gma-miR1507a | BG507325 | NBS-LRR type disease resistance protein RPG1-B |
| gma-miR1507a | NP405782 | NBS-type putative resistance protein |
| gma-miR1507a | TC385742 | Disease resistance-like protein |
| gma-miR1507a | BU084315 | Hypothesis protein |
| gma-miR1507a | TC352643 | Hypothesis protein |
| gma-miR1507a | TC354256 | Hypothesis protein |
| gma-miR1507a | TC417541 | Hypothesis protein |
| gma-miR1508a | TC369712 | Hypothesis protein |
| gma-miR1508a | TC354458 | Glycine max clone GMFP1 isoprenylated protein |
| gma-miR1508a | GD865748 | Hypothesis protein |
| gma-miR1508a | TC356614 | Hypothesis protein |
| gma-miR1508a | DB981540 | Hypothesis protein |
| gma-miR1508a | GD990999 | Hypothesis protein |
| gma-miR1508a | TC383775 | Calcium dependent protein kinase 3 |
| gma-miR1508a | TC419009 | Hypothesis protein |
| gma-miR1508a | TC364594 | Calcium-dependent protein kinase |
| gma-miR1508a | TC399445 | T7A14.6 protein |
| gma-miR1508a | TC399889 | BZIP transcription factor bZIP68 |
| gma-miR1508a | FG997987 | C-type cytochrome biogenesis protein |
| gma-miR1508a | TC416380 | Hypothesis protein |
| gma-miR1508a | TC391618 | Hypothesis protein |
| gma-miR1508a | TC397382 | Hypothesis protein |
| gma-miR1508a | TC367904 | Hypothesis protein |
| gma-miR1508a | TC386174 | Hypothesis protein |
| gma-miR1508a | TC396902 | Zinc finger, C2H2-type |
| gma-miR1508a | TC384041 | PRP38 family protein |
| gma-miR1509a | TC363080 | hypothesis protein |
| gma-miR1509a | BM308337 | Hypothesis protein |
| gma-miR1509a | TC368053 | Hypothesis protein |
| gma-miR1509a | BI785214 | Hypothesis protein |
| gma-miR1509a | TC386520 | Hypothesis protein |
| gma-miR1509a | TC370159 | Hypothesis protein |
| gma-miR1509a | TC364226 | Hypothesis protein |
| gma-miR1509a | TC373728 | Hypothesis protein |
| gma-miR1509a | FK020543 | Mevalonate pyrophosphate decarboxylase |
| gma-miR1509a | TC371496 | Hypothesis protein |
| gma-miR1510a-3p | TC369625 | Functional candidate resistance protein KR1 |
| gma-miR1510a-3p | TC372980 | R 1 protein |
| gma-miR1510a-3p | TC373286 | Resistance protein MG63 |
| gma-miR1510a-3p | TC381520 | Resistance protein PLTR |
| gma-miR1510a-3p | TC369804 | resistance-like protein KNBS1 |
| gma-miR1510a-3p | NP8360889 | ATP synthase beta subunit |
| gma-miR1510a-3p | NP8360891 | ATP synthase beta subunit |
| gma-miR1510a-3p | TC348709 | NBS-LRR type disease resistance protein RPG1-B |
| gma-miR1510a-3p | TC417078 | Disease resistance-like protein |
| gma-miR1510a-3p | BU546011 | Hypothesis protein |
| gma-miR1510a-3p | TC376082 | NBS-LRR disease resistance-like protein |
| gma-miR1510a-3p | TC378443 | Disease resistance protein |
| gma-miR1510a-3p | TC354203 | Resistance protein LM12 |
| gma-miR1510a-3p | TC348768 | ATP synthase subunit beta |
| gma-miR1510a-3p | TC381260 | Hypothesis protein |
| gma-miR1510a-3p | TC360135 | Hypothesis protein |
| gma-miR1510a-3p | TC352578 | Hypothesis protein |
| gma-miR1510a-3p | TC417660 | Hypothesis protein |
| gma-miR1510a-3p | TC372266 | Hypothesis protein |
| gma-miR1510a-5p | BU544584 | Hypothesis protein |
| gma-miR1510a-5p | TC386592 | Hypothesis protein |
| gma-miR1510a-5p | TC415285 | Hypothesis protein |
| gma-miR1510a-5p | CF807079 | Ig domain protein group 2 domain protein precursor |
| gma-miR1510a-5p | TC372705 | Hypothesis protein |
| gma-miR1510a-5p | TC358485 | Ferrochelatase |
| gma-miR1510a-5p | TC371263 | Hypothesis protein |
| gma-miR1510a-5p | CA802529 | Hypothesis protein |
| gma-miR1510a-5p | TC376460 | RanBP2-type zinc finger protein At1g67325 |
| gma-miR1510a-5p | TC376296 | Hypothesis protein |
| gma-miR1510a-5p | TC375999 | Hypothesis protein |
| gma-miR1510a-5p | TC401295 | Ras GTPase |
| gma-miR1510a-5p | FK451740 | Hypothesis protein |
| gma-miR1510a-5p | TC376397 | Hypothesis protein |
| gma-miR1510a-5p | TC397950 | Ras GTPase; Sigma-54 factor, interaction region |
| gma-miR1510a-5p | TC413318 | NAD(+) kinase |
| gma-miR1510a-5p | TC377923 | Hypothesis protein |
| gma-miR1510a-5p | TC350119 | Hypothesis protein |
| gma-miR1510a-5p | TC370673 | Hypothesis protein |
| gma-miR1512 | TC373665 | Hypothesis protein |
| gma-miR1512 | TC358512 | Uncharacterized protein At3g15840.3 |
| gma-miR1512 | TC363401 | T8K14.20 protein |
| gma-miR1512 | TC380995 | Metalloendopeptidase |
| gma-miR1512 | TC355801 | T8K14.20 protein |
| gma-miR1512 | TC377074 | T8K14.20 protein |
| gma-miR1512 | TC389503 | F-box protein interaction domain |
| gma-miR1512 | TC382314 | Hypothesis protein |
| gma-miR1512 | BM893207 | LOB domain-containing protein 41 |
| gma-miR1512 | GD916278 | tRNA (mo5U34)-methyltransferase |
| gma-miR1512 | FK313535 | Hypothesis protein |
| gma-miR1512 | BI316979 | Hypothesis protein |
| gma-miR1512 | TC410527 | Hypothesis protein |
| gma-miR1512 | TC415493 | MADS transcription factor |
| gma-miR1512 | TC418180 | NADH dehydrogenase subunit F |
| gma-miR1512 | AW458784 | Hypothesis protein |
| gma-miR1512 | TC388681 | Hypothesis protein |
| gma-miR1515 | TC355542 | Hypothesis protein |
| gma-miR1515 | GD823852 | Not CCR4-Not complex component, N-terminal; tRNA-binding arm |
| gma-miR1515 | TC412336 | Not CCR4-Not complex component, N-terminal; tRNA-binding arm |
| gma-miR1515 | TC379972 | Aquaporin 2 |
| gma-miR1515 | TC364217 | Pip1 protein |
| gma-miR1515 | TC362601 | Hypothesis protein |
| gma-miR1515 | TC388258 | trihelix transcription factor |
| gma-miR1515 | AW101912 | Hypothesis protein |
| gma-miR1515 | GR827086 | Hypothesis protein |
| gma-miR1517 | TC392990 | Hypothesis protein |
| gma-miR1517 | TC396790 | Hypothesis protein |
| gma-miR1517 | TC410056 | Hypothesis protein |
| gma-miR1517 | TC356493 | HhH-GPD base excision DNA repair family protein |
| gma-miR1517 | TC374318 | Hypothesis protein |
| gma-miR1517 | TC416681 | Hypothesis protein |
| gma-miR1517 | TC392570 | APETAL2-like protein |
| gma-miR1520d | GD932501 | Hypothesis protein |
| gma-miR1520d | TC381076 | Hypothesis protein |
| gma-miR1520d | BU081802 | Hypothesis protein |
| gma-miR1520d | BM567767 | Hypothesis protein |
| gma-miR1520d | TC418103 | Peptidyl-prolyl isomerase FKBP12 |
| gma-miR1520d | DB966959 | Coat protein |
| gma-miR1520d | FK298459 | Predicted protein |
| gma-miR1520d | TC369085 | Hypothesis protein |
| gma-miR1520d | BE474695 | Hypothesis protein |
| gma-miR1520d | TC405300 | Hypothesis protein |
| gma-miR1520d | TC377524 | Hypothesis protein |
| gma-miR1520d | TC377470 | Guanine nucleotide-binding protein alpha-2 subunit |
| gma-miR1520e | TC381076 | Hypothesis protein |
| gma-miR1520e | TC374109 | Hypothesis protein |
| gma-miR1520e | GD932501 | Hypothesis protein |
| gma-miR1520e | TC374493 | Hypothesis protein |
| gma-miR1520e | BM142909 | Intracellular septation protein A |
| gma-miR1520e | GE010994 | Hypothesis protein |
| gma-miR1520e | TC386729 | Hypothesis protein |
| gma-miR1520f | TC381076 | Hypothesis protein |
| gma-miR1520f | GD932501 | Hypothesis protein |
| gma-miR1520f | TC405098 | Hypothesis protein |
| gma-miR1520f | GD989348 | Hypothesis protein |
| gma-miR1520f | FK298459 | Predicted protein |
| gma-miR1520f | BI425292 | Hypothesis protein |
| gma-miR1520f | FK616125 | Hypothesis protein |
| gma-miR1520f | TC402290 | 40S ribosomal protein S30-like |
| gma-miR1520f | BM187655 | Cytochrome c oxidase subunit I |
| gma-miR1520g | TC381076 | Hypothesis protein |
| gma-miR1520g | GD932501 | Hypothesis protein |
| gma-miR1520g | TC405098 | Hypothesis protein |
| gma-miR1520g | GD989348 | Hypothesis protein |
| gma-miR1520g | FK298459 | Predicted protein |
| gma-miR1520g | TC399407 | Hypothesis protein |
| gma-miR1520g | TC410825 | Hypothesis protein |
| gma-miR1520g | TC383625 | Hypothesis protein |
| gma-miR1520g | TC358951 | Hypothesis protein |
| gma-miR1520h | GD932501 | Hypothesis protein |
| gma-miR1520h | TC417740 | Hypothesis protein |
| gma-miR1520h | BF424867 | Hypothesis protein |
| gma-miR1520h | TC409578 | Hypothesis protein |
| gma-miR1520i | TC381076 | Hypothesis protein |
| gma-miR1520i | TC374109 | Hypothesis protein |
| gma-miR1520i | GD932501 | Hypothesis protein |
| gma-miR1520i | TC353422 | Hypothesis protein |
| gma-miR1520i | TC359106 | Hypothesis protein |
| gma-miR1520i | TC386142 | Hypothesis protein |
| gma-miR1520j | TC381076 | Hypothesis protein |
| gma-miR1520j | TC363411 | Hypothesis protein |
| gma-miR1520j | TC374109 | Hypothesis protein |
| gma-miR1520j | TC412727 | U1snRNP-specific protein, U1A |
| gma-miR1520j | TC381011 | Hypothesis protein |
| gma-miR1520j | TC418103 | Peptidyl-prolyl isomerase FKBP12 |
| gma-miR1520k | GD932501 | Hypothesis protein |
| gma-miR1520k | TC381076 | Hypothesis protein |
| gma-miR1520k | TC369085 | Hypothesis protein |
| gma-miR1520k | DB966959 | Coat protein |
| gma-miR1520k | TC381566 | Hypothesis protein |
| gma-miR1520k | FK616125 | Hypothesis protein |
| gma-miR1520k | GD989348 | Hypothesis protein |
| gma-miR1520k | BM187655 | Cytochrome c oxidase subunit I |
| gma-miR1520k | TC402290 | 40S ribosomal protein S30-like |
| gma-miR1520k | TC405098 | Hypothesis protein |
| gma-miR1520l | GD932501 | Hypothesis protein |
| gma-miR1520l | TC381076 | Hypothesis protein |
| gma-miR1520l | FG991890 | Hypothesis protein |
| gma-miR1520l | DB989008 | Glyoxal or galactose oxidase |
| gma-miR1520l | TC389647 | Hypothesis protein |
| gma-miR1520l | GD989348 | Hypothesis protein |
| gma-miR1520m | GD932501 | Hypothesis protein |
| gma-miR1520m | TC381076 | Hypothesis protein |
| gma-miR1520m | BI425292 | Hypothesis protein |
| gma-miR1520n | TC381076 | Hypothesis protein |
| gma-miR1520n | GD932501 | Hypothesis protein |
| gma-miR1520n | FG991890 | Hypothesis protein |
| gma-miR1520n | BI425292 | Hypothesis protein |
| gma-miR1520o | GD932501 | Hypothesis protein |
| gma-miR1520o | TC394294 | Polygalacturonase-1 non-catalytic subunit beta precursor |
| gma-miR1520o | TC381285 | Hypothesis protein |
| gma-miR1520o | TC357470 | Polygalacturonase-1 non-catalytic subunit beta precursor |
| gma-miR1520o | BF066563 | Os01g0658700 protein |
| gma-miR1520o | TC419078 | Fasciclin-like arabinogalactan protein 10 |
| gma-miR1520o | TC365330 | Hypothesis protein |
| gma-miR1520o | TC360091 | Hypothesis protein |
| gma-miR1520o | TC382570 | Hexokinase 6 |
| gma-miR1520o | TC350793 | Hypothesis protein |
| gma-miR1520o | TC415799 | Hypothesis protein |
| gma-miR1520o | TC388592 | Hypothesis protein |
| gma-miR1520p | TC393546 | Hypothesis protein |
| gma-miR1520p | FK515825 | Hypothesis protein |
| gma-miR1520p | DB956505 | Homeobox protein otx |
| gma-miR1520p | BE209422 | U2 auxiliary factor small subunit |
| gma-miR1520p | TC416370 | Hypothesis protein |
| gma-miR1520p | TC395705 | Predicted protein |
| gma-miR1520p | TC411285 | Ribosomal protein L19 |
| gma-miR1520p | TC351561 | Sodium-driven multidrug efflux pump |
| gma-miR1520p | BQ296653 | Hypothesis protein |
| gma-miR1520p | BE822862 | 2'-hydroxy isoflavone/dihydroflavonol reductase homolog |
| gma-miR1520p | EV272008 | S-domain receptor-like protein kinase precursor |
| gma-miR1520p | CD416569 | Sensor protein |
| gma-miR1520p | TC375912 | Hypothesis protein |
| gma-miR1520p | TC368399 | Hypothesis protein |
| gma-miR1520p | TC383886 | Hypothesis protein |
| gma-miR1520p | TC352794 | Hypothesis protein |
| gma-miR1520p | TC372315 | Dof1 |
| gma-miR1520q | TC391996 | Os06g0186900 protein |
| gma-miR1520q | TC358444 | Os06g0186900 protein |
| gma-miR1520q | TC412645 | Hypothesis protein |
| gma-miR1520q | TC417358 | Calcium-dependent protein kinase SK5 |
| gma-miR1520q | GE138239 | Hypothesis protein |
| gma-miR1520q | TC395421 | Hypothesis protein |
| gma-miR1520q | TC399842 | YT521-B-like protein |
| gma-miR1520q | TC383015 | Sucrose transport protein |
| gma-miR1520q | TC364052 | Hypothesis protein |
| gma-miR1520q | TC419549 | Hypothesis protein |
| gma-miR1520q | TC355374 | Hypothesis protein |
| gma-miR1520q | FG991645 | Hypothesis protein |
| gma-miR1520q | TC379703 | Hypothesis protein |
| gma-miR1520q | TC373162 | Hypothesis protein |
| gma-miR1520q | TC353087 | Hypothesis protein |
| gma-miR1520q | TC419788 | G-box binding factor |
| gma-miR1520q | TC404859 | G-box binding factor |
| gma-miR1520r | TC365328 | Transport inhibitor response 1 |
| gma-miR1520r | TC362546 | Transport inhibitor response 1 |
| gma-miR1526 | TC367846 | Guanine nucleotide regulatory protein |
| gma-miR1526 | TC357653 | Adenosylhomocysteinase |
| gma-miR1526 | TC409556 | Adenosylhomocysteinase |
| gma-miR1526 | TC374036 | Histone H4 |
| gma-miR1526 | TC372507 | Adenosylhomocysteinase |
| gma-miR1526 | DB986637 | Adenosylhomocysteinase |
| gma-miR1526 | TC363265 | Hypothesis protein |
| gma-miR1526 | AW705307 | N3 protein |
| gma-miR1526 | GD870962 | Predicted protein |
| gma-miR1526 | TC394574 | SINA6 |
| gma-miR1526 | TC354465 | Hypothesis protein |
| gma-miR1526 | TC357205 | Hypothesis protein |
| gma-miR1526 | TC373845 | Uncharacterized protein At2g35230.2 |
| gma-miR1526 | TC357031 | Nucleic acid binding related |
| gma-miR1526 | FK521947 | Hypothesis protein |
| gma-miR1526 | TC405259 | Hypothesis protein |
| gma-miR1526 | TC396185 | Hypothesis protein |
| gma-miR1526 | TC396241 | Glucose-1-phosphate adenylyltransferase |
| gma-miR1526 | TC416212 | Hypothesis protein |
| gma-miR1526 | TC375322 | Hypothesis protein |
| gma-miR1526 | GD661098 | Hypothesis protein |
| gma-miR1526 | TC388729 | Hypothesis protein |
| gma-miR1526 | TC350380 | Ferrous ion membrane transport protein DMT1 |
| gma-miR1531 | TC355586 | Hypothesis protein |
| gma-miR1531 | TC351854 | Hypothesis protein |
| gma-miR1531 | BI316933 | Hypothesis protein |
| gma-miR1531 | TC379893 | 26S protease regulatory subunit 6B homolog |
| gma-miR1531 | BE347472 | Hypothesis protein |
| gma-miR1531 | TC384567 | Hypothesis protein |
| gma-miR1531 | TC350256 | Hypothesis protein |
| gma-miR1531 | GD973189 | Starch branching enzyme I |
| gma-miR1531 | AW279267 | Senescence-inducible chloroplast stay-green protein 1 |
| gma-miR1863 | TC369348 | Helix-turn-helix motif:Peptidase S24, S26A and S26B |
| gma-miR1863 | CO986013 | Hypothesis protein |
| gma-miR1863 | CD407892 | Tic62 protein precursor |
| gma-miR1863 | CD397679 | Tic62 protein precursor |
| gma-miR1863 | TC370881 | Probable serine/threonine-protein kinase GCN2 |
| gma-miR1863 | EV270504 | Glucan endo-1,3-beta-glucosidase |
| gma-miR1863 | TC359601 | Glucan endo-1,3-beta-glucosidase |
| gma-miR1863 | TC360468 | Glucan endo-1,3-beta-glucosidase |
| gma-miR1863 | TC371923 | Predicted protein |
| gma-miR1863 | AW757016 | Hypothesis protein |
| gma-miR1863 | TC386641 | Hypothesis protein |
| gma-miR1863 | TC373485 | Hypothesis protein |
| gma-miR1863 | FG989950 | Outer membrane protein Haemagluttinin-like |
| gma-miR1863 | TC380812 | Hypothesis protein |
| gma-miR1863 | TC351084 | 1,3-beta-D-glucanase |
| gma-miR1863 | AW705411 | MAP kinase PsMAPK2 |
| gma-miR1863 | EV265155 | Hypothesis protein |
| gma-miR1863 | TC359929 | transporter-related |
| gma-miR1863 | EV270285 | Hypothesis protein |
| gma-miR1863 | BE800269 | Hypothesis protein |
| gma-miR1863 | BM528293 | Hypothesis protein |
| gma-miR1863 | TC373048 | Hypothesis protein |
| gma-miR1863 | TC352093 | Os04g0137500 protein |
| gma-miR1863 | TC370534 | Choline acetyltransferase |
| gma-miR2089 | EH261005 | elastin b |
| gma-miR2089 | TC373286 | Resistance protein MG63 |
| gma-miR2089 | TC358405 | Nodulin-like protein |
| gma-miR2089 | TC360871 | Hypothesis protein |
| gma-miR2089 | TC369625 | Functional candidate resistance protein KR1 |
| gma-miR2089 | TC368975 | Hypothesis protein |
| gma-miR2089 | TC360517 | Hypothesis protein |
| gma-miR2089 | TC395494 | NBS-LRR-Toll resistance gene analog protein |
| gma-miR2089 | TC419808 | Resistance protein PLTR |
| gma-miR2089 | TC358536 | Resistance protein KR3 |
| gma-miR2089 | TC390975 | Hypothesis protein |
| gma-miR2089 | GD780042 | AT3g53540/F4P12_240 |
| gma-miR2089 | BE021239 | Xylem serine proteinase 1 precursor |
| gma-miR2118 | TC404387 | Hypothesis protein |
| gma-miR2118 | TC405437 | Hypothesis protein |
| gma-miR2118 | GE124754 | Hypothesis protein |
| gma-miR2118 | GD838207 | Hypothesis protein |
| gma-miR2118 | BI973678 | Hypothesis protein |
| gma-miR2118 | TC385797 | Hypothesis protein |
| gma-miR2118 | TC365674 | Hypothesis protein |
| gma-miR2118 | TC388562 | Hypothesis protein |
| gma-miR2118 | TC367553 | Hypothesis protein |
| gma-miR2118 | TC348709 | NBS-LRR type disease resistance protein RPG1-B |
| gma-miR2118 | TC350379 | Hypothesis protein |
| gma-miR2118 | AW201204 | Os03g0411900 protein |
| gma-miR2118 | TC395494 | NBS-LRR-Toll resistance gene analog protein |
| gma-miR2118 | TC387772 | disease resistance-like protein |
| gma-miR2118 | TC409747 | Probable pectate lyase 12 precursor |
| gma-miR2118 | TC419808 | Resistance protein PLTR |
| gma-miR3522b | TC380602 | Polyphenol oxidase |
| gma-miR3522b | BW668066 | Laminin subunit beta-1 variant |
| gma-miR3522b | CD399306 | Hypothesis protein |
| gma-miR3522b | TC359036 | Hypothesis protein |
| gma-miR3522b | TC370429 | flavonoid glycosyltransferase |
| gma-miR3522b | TC406038 | Heavy metal transporter MTP1 |
| gma-miR3522b | AW705218 | Hypothesis protein |
| gma-miR4340 | EH262489 | ATP synthase beta chain |
| gma-miR4340 | DB968923 | Magnesium-chelatase subunit chlD |
| gma-miR4340 | TC382500 | Magnesium-chelatase subunit chlD |
| gma-miR4340 | TC388942 | GRAS10 |
| gma-miR4340 | TC356792 | Protein kinase 5 |
| gma-miR4340 | TC363647 | Protein kinase 5 |
| gma-miR4341 | TC407183 | Cold regulated 413 plasma membrane 1 |
| gma-miR4341 | CO982670 | Hypothesis protein |
| gma-miR4341 | TC412387 | Hypothesis protein |
| gma-miR4341 | FK018432 | Glycoside hydrolase |
| gma-miR4341 | BG882907 | Hypothesis protein |
| gma-miR4341 | TC388416 | Hypothesis protein |
| gma-miR4341 | BW651121 | Hypothesis protein |
| gma-miR4341 | EV273066 | Hypothesis protein |
| gma-miR4341 | TC381404 | Hypothesis protein |
| gma-miR4341 | AW782057 | Hypothesis protein |
| gma-miR4341 | GE091097 | Hypothesis protein |
| gma-miR4341 | FK528982 | Hypothesis protein |
| gma-miR4343a | TC397839 | Hypothesis protein |
| gma-miR4343a | TC355239 | Hypothesis protein |
| gma-miR4343a | TC366322 | Hypothesis protein |
| gma-miR4343a | TC378003 | Hypothesis protein |
| gma-miR4343a | TC378890 | Hypothesis protein |
| gma-miR4343a | EV268647 | Mitotic cyclin a1-type |
| gma-miR4343a | TC356005 | NR1 |
| gma-miR4343b | TC377199 | RAB1Y |
| gma-miR4343b | EH221327 | Ribosomal protein L15 |
| gma-miR4343b | BI321091 | RAD23-like |
| gma-miR4343b | GD685639 | Hypothesis protein |
| gma-miR4343b | BW680390 | Predicted protein |
| gma-miR4343b | TC407697 | Hypothesis protein |
| gma-miR4343b | TC369710 | Hypothesis protein |
| gma-miR4343b | CA937717 | Predicted protein |
| gma-miR4343b | AW278855 | Os02g0581400 protein |
| gma-miR4343b | TC357132 | Predicted protein |
| gma-miR4343b | TC389582 | Histone H2A |
| gma-miR4343b | TC375782 | Hypothesis protein |
| gma-miR4344 | TC361897 | Predicted protein |
| gma-miR4344 | TC387564 | Hypothesis protein |
| gma-miR4344 | TC371840 | Hypothesis protein |
| gma-miR4344 | TC397964 | Hypothesis protein |
| gma-miR4344 | TC396472 | Hypothesis protein |
| gma-miR4344 | TC354225 | Hypothesis protein |
| gma-miR4344 | TC410975 | Hypothesis protein |
| gma-miR4344 | TC360306 | Hypothesis protein |
| gma-miR4345 | TC414444 | 110 kDa 4SNc-Tudor domain protein |
| gma-miR4345 | TC353004 | 110 kDa 4SNc-Tudor domain protein |
| gma-miR4345 | EV267973 | Hypothesis protein |
| gma-miR4345 | TC362773 | 110 kDa 4SNc-Tudor domain protein |
| gma-miR4345 | BU084118 | Hypothesis protein |
| gma-miR4345 | TC374823 | Hypothesis protein |
| gma-miR4345 | BW653222 | Hypothesis protein |
| gma-miR4345 | TC404309 | procollagen-proline |
| gma-miR4345 | TC375574 | Hypothesis protein |
| gma-miR4345 | TC377648 | Actin-like protein |
| gma-miR4345 | TC354249 | NADP-specific isocitrate dehydrogenase |
| gma-miR4345 | BF069235 | Hypothesis protein |
| gma-miR4345 | TC419936 | Proteasome subunit alpha type-2-A |
| gma-miR4347 | DB975263 | Hypothesis protein |
| gma-miR4347 | TC356076 | Hypothesis protein |
| gma-miR4347 | TC364434 | Probable histone-arginine methyltransferase CARM1 |
| gma-miR4347 | TC395858 | Hypothesis protein |
| gma-miR4347 | EH224342 | DNA mismatch repair protein MutS |
| gma-miR4347 | TC353453 | Hypothesis protein |
| gma-miR4347 | AW200882 | Hypothesis protein |
| gma-miR4347 | TC356388 | F-box protein AtFBL5 |
| gma-miR4347 | BE346210 | Glucose-1-phosphate adenylyltransferase |
| gma-miR4349 | TC369487 | Katanin-like protein |
| gma-miR4349 | TC384041 | PRP38 family protein |
| gma-miR4349 | AW349561 | Hypothesis protein |
| gma-miR4349 | TC398049 | Hypothesis protein |
| gma-miR4349 | DB979838 | Hypothesis protein |
| gma-miR4351 | TC415493 | MADS transcription factor |
| gma-miR4351 | TC415975 | MADS transcription factor |
| gma-miR4351 | TC394140 | MADS box protein SEP3 |
| gma-miR4351 | BI425185 | Uncharacterized protein YDR461C-A |
| gma-miR4351 | TC415166 | Hypothesis protein |
| gma-miR4351 | TC382388 | Hypothesis protein |
| gma-miR4351 | TC391804 | MADS transcription factor |
| gma-miR4351 | TC381453 | Expressed protein |
| gma-miR4351 | BE803966 | Hypothesis protein |
| gma-miR4351 | TC351050 | WRKY62 |
| gma-miR4351 | TC376946 | Hypothesis protein |
| gma-miR4351 | TC396838 | Hypothesis protein |
| gma-miR4351 | TC419135 | Hypothesis protein |
| gma-miR4351 | TC356353 | Hypothesis protein |
| gma-miR4351 | TC406462 | MADS box protein SEP3 |
| gma-miR4351 | GD978340 | PVR3-like protein |
| gma-miR4351 | TC414424 | Hypothesis protein |
| gma-miR4351 | TC350789 | Hypothesis protein |
| gma-miR4352a | TC414025 | TRAP dicarboxylate transporter |
| gma-miR4352a | TC348889 | Hypothesis protein |
| gma-miR4352a | TC388827 | Sinapoylglucose:choline sinapoyltransferase |
| gma-miR4352a | BM309580 | Cytochrome b |
| gma-miR4352a | BE820323 | Hypothesis protein |
| gma-miR4352b | BM309671 | Hypothesis protein |
| gma-miR4352b | TC360951 | Hypothesis protein |
| gma-miR4352b | TC384937 | Hypothesis protein |
| gma-miR4352b | GE018191 | Tubulin alpha-1 chain |
| gma-miR4353 | BU762717 | Hypothesis protein |
| gma-miR4353 | TC360722 | Hypothesis protein |
| gma-miR4353 | TC367609 | Hypothesis protein |
| gma-miR4353 | TC392350 | Hypothesis protein |
| gma-miR4353 | TC420598 | Hypothesis protein |
| gma-miR4357 | BF009193 | Sulfate adenylyltransferase |
| gma-miR4357 | AW186199 | Hypothesis protein |
| gma-miR4357 | TC360434 | Hypothesis protein |
| gma-miR4357 | BM086465 | Hypothesis protein |
| gma-miR4357 | TC373854 | Hypothesis protein |
| gma-miR4358 | FK478334 | Hypothesis protein |
| gma-miR4358 | TC408722 | Hypothesis protein |
| gma-miR4359a | TC352184 | Hypothesis protein |
| gma-miR4359a | TC397873 | Hypothesis protein |
| gma-miR4359b | GD866244 | Hypothesis protein |
| gma-miR4359b | EV271848 | UOS1 |
| gma-miR4359b | TC352083 | Hypothesis protein |
| gma-miR4359b | TC355871 | Hypothesis protein |
| gma-miR4359b | TC373649 | Hypothesis protein |
| gma-miR4360 | FK447349 | Hypothesis protein |
| gma-miR4360 | BF324330 | Hypothesis protein |
| gma-miR4360 | TC378357 | Hypothesis protein |
| gma-miR4360 | TC404764 | Hypothesis protein |
| gma-miR4361 | TC395840 | Hypothesis protein |
| gma-miR4361 | TC393875 | Hypothesis protein |
| gma-miR4361 | GD927609 | Hypothesis protein |
| gma-miR4361 | TC414929 | Hypothesis protein |
| gma-miR4363 | TC362053 | Hypothesis protein |
| gma-miR4363 | TC356771 | Hypothesis protein |
| gma-miR4363 | FG988628 | Hypothesis protein |
| gma-miR4363 | TC376830 | Glycosyl transferase, family 48 |
| gma-miR4364a | TC411245 | 1-deoxy-D-xylulose 5-phosphate synthase 2 precursor |
| gma-miR4364a | TC355369 | Hypothesis protein |
| gma-miR4364a | FK370239 | Hypothesis protein |
| gma-miR4364a | TC396146 | Hypothesis protein |
| gma-miR4364a | GD836392 | Hypothesis protein |
| gma-miR4364a | TC398011 | Hypothesis protein |
| gma-miR4364b | TC398353 | Hypothesis protein |
| gma-miR4364b | TC366295 | Hypothesis protein |
| gma-miR4364b | TC380573 | Hypothesis protein |
| gma-miR4364b | CX704197 | Hypothesis protein |
| gma-miR4364b | TC420637 | Hypothesis protein |
| gma-miR4364b | TC393146 | 40S ribosomal protein S23 |
| gma-miR4364b | TC399976 | Hypothesis protein |
| gma-miR4364b | TC378896 | Dopamine receptor, D1 |
| gma-miR4364b | TC360577 | Trypsin inhibitor |
| gma-miR4365 | TC389837 | Hypothesis protein |
| gma-miR4365 | TC373755 | 3'-5' exonuclease domain-containing protein-like |
| gma-miR4365 | TC412585 | Targeting protein for Xklp2 containing protein |
| gma-miR4365 | TC360396 | Hypothesis protein |
| gma-miR4365 | TC350494 | WRKY17 |
| gma-miR4365 | BE023717 | Hypothesis protein |
| gma-miR4365 | TC391953 | Hypothesis protein |
| gma-miR4366 | TC419399 | Hypothesis protein |
| gma-miR4366 | TC419925 | Hypothesis protein |
| gma-miR4366 | TC399665 | CDC2 |
| gma-miR4366 | TC355466 | Hypothesis protein |
| gma-miR4366 | TC379001 | Hypothesis protein |
| gma-miR4366 | BM188318 | Hypothesis protein |
| gma-miR4366 | TC413305 | Hypothesis protein |
| gma-miR4366 | CD410470 | Hypothesis protein |
| gma-miR4366 | FK279920 | Hypothesis protein |
| gma-miR4366 | TC402088 | Hypothesis protein |
| gma-miR4366 | BM528484 | Hypothesis protein |
| gma-miR4367 | TC420215 | Hypothesis protein |
| gma-miR4367 | TC389978 | Hypothesis protein |
| gma-miR4367 | EV280528 | Hypothesis protein |
| gma-miR4367 | TC362023 | Hypothesis protein |
| gma-miR4367 | TC379233 | AP2/EREBP transcription factor |
| gma-miR4367 | TC401462 | Hypothesis protein |
| gma-miR4367 | GD888720 | Hypothesis protein |
| gma-miR4368b | TC415580 | BH3550 protein |
| gma-miR4368b | TC398900 | Hypothesis protein |
| gma-miR4368b | TC391662 | Hypothesis protein |
| gma-miR4368b | TC402720 | Hypothesis protein |
| gma-miR4368b | TC411281 | Hypothesis protein |
| gma-miR4369 | BU544553 | ABC-type bacteriocin/lantibiotic exporters |
| gma-miR4369 | BW664591 | Hypothesis protein |
| gma-miR4369 | TC364430 | Hypothesis protein |
| gma-miR4369 | FK430414 | Dehydration-induced protein |
| gma-miR4369 | TC412367 | Hypothesis protein |
| gma-miR4369 | FK316596 | Dehydration-induced protein |
| gma-miR4369 | GD689867 | Hypothesis protein |
| gma-miR4369 | GD754448 | Hypothesis protein |
| gma-miR4369 | TC388247 | Dehydration-induced protein |
| gma-miR4369 | GD865480 | Dehydration-induced protein |
| gma-miR4369 | GD811886 | Hypothesis protein |
| gma-miR4369 | GD825224 | Hypothesis protein |
| gma-miR4369 | TC388783 | Dehydration-induced protein |
| gma-miR4369 | TC368056 | Multiple antibiotic transporter |
| gma-miR4369 | FG993867 | Dehydration-induced protein |
| gma-miR4369 | DY577159 | Peroxidase precursor |
| gma-miR4369 | TC397026 | Dehydration-induced protein |
| gma-miR4370 | BI317600 | Na+/H+ antiporter |
| gma-miR4370 | TC367091 | Hypothesis protein |
| gma-miR4370 | BW671403 | Hypothesis protein |
| gma-miR4370 | TC411103 | Transcription initiation factor |
| gma-miR4370 | TC370659 | Hypothesis protein |
| gma-miR4370 | TC359021 | Beta-fructofuranosidase; cell wall invertase I; fructosidase |
| gma-miR4370 | TC364029 | Hypothesis protein |
| gma-miR4370 | TC378159 | Hypothesis protein |
| gma-miR4370 | TC374750 | Hypothesis protein |
| gma-miR4370 | TC379847 | Hypothesis protein |
| gma-miR4370 | TC388059 | Hypothesis protein |
| gma-miR4370 | TC359489 | Hypothesis protein |
| gma-miR4371a | EH260467 | Hypothesis protein |
| gma-miR4371a | TC416419 | Hypothesis protein |
| gma-miR4371a | TC400742 | Isocitrate dehydrogenase [NADP] |
| gma-miR4371a | TC351786 | Isocitrate dehydrogenase [NADP] |
| gma-miR4371b | CO984976 | Hypothesis protein |
| gma-miR4371b | TC365592 | Hypothesis protein |
| gma-miR4371b | BG315903 | Transcription factor |
| gma-miR4371c | TC363156 | Hypothesis protein |
| gma-miR4371c | FK401778 | Hypothesis protein |
| gma-miR4373 | FK447349 | Hypothesis protein |
| gma-miR4373 | BF324330 | Hypothesis protein |
| gma-miR4373 | TC356207 | Multidrug resistance protein 1 |
| gma-miR4373 | TC404764 | Hypothesis protein |
| gma-miR4373 | TC378357 | Hypothesis protein |
| gma-miR4374a | BF070260 | Methylenetetrahydrofolate reductase |
| gma-miR4374a | TC365048 | Secretory carrier-associated membrane protein |
| gma-miR4374a | TC392455 | Hypothesis protein |
| gma-miR4374a | TC368092 | Glutamine synthetase cytosolic isozyme 1 |
| gma-miR4374a | TC401803 | cytosolic glutamine synthetase beta2 |
| gma-miR4374a | TC352275 | Glutamine synthetase cytosolic isozyme 1 |
| gma-miR4374a | TC406449 | Glutamine synthetase nodule isozyme |
| gma-miR4374a | TC417607 | CPRD12 protein |
| gma-miR4374a | TC353250 | Cytosolic glutamine synthetase GSbeta1 |
| gma-miR4374a | TC386106 | Cytosolic glutamine synthetase GSbeta1 |
| gma-miR4374a | TC350369 | Cytosolic glutamine synthetase GSbeta1 |
| gma-miR4375 | BQ741193 | Hypothesis protein |
| gma-miR4375 | TC403578 | Hypothesis protein |
| gma-miR4376 | TC360394 | Betaine-aldehyde dehydrogenase |
| gma-miR4376 | TC396530 | Betaine-aldehyde dehydrogenase |
| gma-miR4376 | BW676206 | Hypothesis protein |
| gma-miR4376 | TC379476 | Hypothesis protein |
| gma-miR4377 | TC409332 | Hypothesis protein |
| gma-miR4377 | FK297823 | Hypothesis protein |
| gma-miR4377 | FK490485 | Hypothesis protein |
| gma-miR4377 | FK461075 | Central motor kinesin 1 |
| gma-miR4377 | TC368694 | Hypothesis protein |
| gma-miR4377 | TC398290 | Central motor kinesin 1 |
| gma-miR4377 | TC389163 | Expressed protein |
| gma-miR4377 | TC412302 | Hypothesis protein |
| gma-miR4377 | NP595166 | GB|AF541963.1|AAO23072.1 R 14 protein |
| gma-miR4378a | BW654569 | Pantothenate kinase 2 |
| gma-miR4378a | TC383619 | Hypothesis protein |
| gma-miR4378a | CD399608 | Predicted protein |
| gma-miR4378a | GD747296 | Hypothesis protein |
| gma-miR4378a | CA802475 | MADS box protein |
| gma-miR4378b | TC374808 | Hypothesis protein |
| gma-miR4378b | TC356910 | Hypothesis protein |
| gma-miR4378b | TC406882 | Hypothesis protein |
| gma-miR4378b | TC372731 | Ferredoxin--NADP reductase, root isozyme |
| gma-miR4378b | BU577481 | Phosphoglucomutase, chloroplast precursor |
| gma-miR4378b | BU083078 | Glucagon-like peptide 2 receptor precursor |
| gma-miR4378b | TC417576 | Papain-like cysteine proteinase |
| gma-miR4378b | TC410293 | Hypothesis protein |
| gma-miR4378b | TC357462 | Calmodulin-related protein |
| gma-miR4378b | TC356055 | Calmodulin-related protein |
| gma-miR4379 | EV273699 | Daf1 protein |
| gma-miR4379 | BF068174 | Hypothesis protein |
| gma-miR4379 | TC419168 | Hypothesis protein |
| gma-miR4379 | TC396212 | Hypothesis protein |
| gma-miR4379 | TC408308 | Hypothesis protein |
| gma-miR4379 | BW669722 | Hypothesis protein |
| gma-miR4379 | TC401641 | Hypothesis protein |
| gma-miR4379 | TC356207 | Multidrug resistance protein 1 |
| gma-miR4380a | TC418337 | Flavanone 3-hydroxylase |
| gma-miR4380a | TC372362 | Hypothesis protein |
| gma-miR4380a | BW673132 | Protein containing C-terminal RING-finger |
| gma-miR4380a | TC358091 | C-terminal zinc-finger |
| gma-miR4380a | BW657856 | S-adenosylmethionine decarboxylase uORF |
| gma-miR4380a | TC388541 | S-adenosylmethionine decarboxylase |
| gma-miR4380a | TC369555 | Hypothesis protein |
| gma-miR4380a | CB063862 | Protein containing C-terminal RING-finger |
| gma-miR4380a | TC399474 | Photosystem II reaction center protein K precursor |
| gma-miR4380a | EV275774 | Hypothesis protein |
| gma-miR4380a | TC360016 | Protein containing C-terminal RING-finger |
| gma-miR4380a | TC398966 | Hypothesis protein |
| gma-miR4380b | TC416607 | Hypothesis protein |
| gma-miR4380b | TC420481 | Hypothesis protein |
| gma-miR4380b | TC417103 | Hypothesis protein |
| gma-miR4380b | TC397211 | Hypothesis protein |
| gma-miR4380b | FK023942 | Hypothesis protein |
| gma-miR4380b | TC381220 | Pro-neuregulin-1 |
| gma-miR4380b | TC400081 | Hypothesis protein |
| gma-miR4380b | BW674817 | Autophagy-related protein 3 |
| gma-miR4380b | TC368711 | Uncharacterized protein At5g23160 |
| gma-miR4381 | TC409500 | Hypothesis protein |
| gma-miR4381 | TC413344 | VTC2-like protein |
| gma-miR4381 | BI700076 | Hypothesis protein |
| gma-miR4381 | TC352460 | Nodulin family protein |
| gma-miR4381 | FK015294 | Hypothesis protein |
| gma-miR4382 | TC395252 | Protein kinase |
| gma-miR4382 | TC382349 | Hypothesis protein |
| gma-miR4382 | TC405604 | Hypothesis protein |
| gma-miR4382 | TC387103 | Hypothesis protein |
| gma-miR4382 | TC375488 | Hypothesis protein |
| gma-miR4382 | FK412762 | Hypothesis protein |
| gma-miR4382 | TC419601 | Hypothesis protein |
| gma-miR4382 | AI900783 | TGF-beta receptor, type I/II extracellular region |
| gma-miR4382 | TC420415 | Hypothesis protein |
| gma-miR4382 | TC382767 | Predicted protein |
| gma-miR4382 | TC350071 | Predicted protein |
| gma-miR4382 | TC365920 | TGF-beta receptor |
| gma-miR4383 | TC412476 | Pyrophosphate-dependent phosphofructo-1-kinase |
| gma-miR4383 | TC394058 | Hypothesis protein |
| gma-miR4383 | TC386854 | Hypothesis protein |
| gma-miR4383 | TC374035 | Hypothesis protein |
| gma-miR4384 | TC368056 | Multiple antibiotic transporter |
| gma-miR4384 | EH221723 | Hypothesis protein |
| gma-miR4384 | EV263409 | Hypothesis protein |
| gma-miR4384 | TC349706 | T-complex protein 1, alpha subunit |
| gma-miR4384 | TC373543 | Hypothesis protein |
| gma-miR4384 | TC354128 | Hypothesis protein |
| gma-miR4384 | TC396144 | Hypothesis protein |
| gma-miR4384 | TC384795 | Hypothesis protein |
| gma-miR4385 | TC374295 | Hypothesis protein |
| gma-miR4385 | TC354774 | 2,4-D inducible glutathione S-transferase |
| gma-miR4385 | TC398950 | Glutathione transferase |
| gma-miR4385 | TC364877 | Hypothesis protein |
| gma-miR4385 | TC375695 | Prephenate dehydratase with ACT region |
| gma-miR4385 | TC362269 | Prephenate dehydratase with ACT region |
| gma-miR4385 | TC401970 | Chalcone synthase 7 |
| gma-miR4385 | TC418174 | Histone H2A |
| gma-miR4386 | TC350467 | Hypothesis protein |
| gma-miR4386 | TC413789 | Hypothesis protein |
| gma-miR4386 | FK276334 | Hypothesis protein |
| gma-miR4386 | TC410985 | 60S ribosomal protein L6 |
| gma-miR4386 | TC350675 | Hypothesis protein |
| gma-miR4386 | TC385211 | Shikimate kinase |
| gma-miR4386 | TC392407 | Ultraviolet-B-repressible protein |
| gma-miR4386 | BI315853 | Hypothesis protein |
| gma-miR4386 | TC382215 | Hypothesis protein |
| gma-miR4387a | TC389163 | Expressed protein |
| gma-miR4387a | TC404297 | Hypothesis protein |
| gma-miR4387a | TC412164 | Tetratricopeptide-like helical |
| gma-miR4387a | TC398260 | Ser/Thr protein kinase |
| gma-miR4387a | TC367861 | Hypothesis protein |
| gma-miR4387a | TC375730 | intracellular transporter |
| gma-miR4387a | TC359698 | intracellular transporter |
| gma-miR4387a | TC352306 | intracellular transporter |
| gma-miR4387b | TC402035 | Hypothesis protein |
| gma-miR4387b | TC368371 | Acyl-[acyl-carrier protein] desaturase |
| gma-miR4387b | TC350581 | Hypothesis protein |
| gma-miR4387b | TC404297 | Hypothesis protein |
| gma-miR4387b | TC389163 | Expressed protein |
| gma-miR4387b | GE047168 | Hypothesis protein |
| gma-miR4387b | TC392529 | Hypothesis protein |
| gma-miR4387b | DB977560 | Asparagine synthetase |
| gma-miR4387b | DB989247 | Asparagine synthetase |
| gma-miR4387b | TC366395 | Asparagine synthetase |
| gma-miR4387c | TC389163 | Expressed protein |
| gma-miR4387c | TC366497 | Hypothesis protein |
| gma-miR4387c | TC370533 | Hypothesis protein |
| gma-miR4387c | TC350838 | 4-coumarate:coenzyme A ligase |
| gma-miR4387c | TC350144 | 4-coumarate:coenzyme A ligase |
| gma-miR4387c | BI317814 | ABC transporter related |
| gma-miR4388 | TC350451 | RNA-dependent RNA polymerase |
| gma-miR4388 | EH038628 | Replicase |
| gma-miR4388 | BM953911 | Hypothesis protein |
| gma-miR4388 | TC389137 | Hypothesis protein |
| gma-miR4388 | FG988040 | Nodulin-like protein |
| gma-miR4388 | TC377767 | Hypothesis protein |
| gma-miR4388 | TC353170 | Hypothesis protein |
| gma-miR4388 | TC414784 | Hypothesis protein |
| gma-miR4388 | TC351312 | Hypothesis protein |
| gma-miR4390 | BE800658 | Hypothesis protein |
| gma-miR4390 | TC365166 | Hypothesis protein |
| gma-miR4391 | TC406874 | Catalase-4 |
| gma-miR4391 | BI974502 | Hypothesis protein |
| gma-miR4391 | TC352797 | Hypothesis protein |
| gma-miR4391 | TC368957 | Hypothesis protein |
| gma-miR4391 | TC395772 | Hypothesis protein |
| gma-miR4391 | GD709670 | Hypothesis protein |
| gma-miR4391 | TC372301 | Hypothesis protein |
| gma-miR4391 | TC410785 | Hypothesis protein |
| gma-miR4393a | TC412208 | Hypothesis protein |
| gma-miR4393a | TC398289 | Hypothesis protein |
| gma-miR4393a | TC354880 | RNA-directed DNA polymerase |
| gma-miR4393a | GD873559 | Two-component system response regulator |
| gma-miR4393a | TC361351 | Hypothesis protein |
| gma-miR4393a | BG652087 | Transcriptional regulator, MerR family |
| gma-miR4393a | TC411091 | Hypothesis protein |
| gma-miR4393a | TC369608 | Hypothesis protein |
| gma-miR4393a | TC358752 | Glycine-rich protein |
| gma-miR4393a | TC406230 | HMG-CoA synthase 2 |
| gma-miR4393b | GE106708 | Hypothesis protein |
| gma-miR4393b | TC349291 | Phosphoserine aminotransferase |
| gma-miR4393b | TC350900 | Hypothesis protein |
| gma-miR4393b | TC370214 | Hypothesis protein |
| gma-miR4393b | TC371604 | Hypothesis protein |
| gma-miR4393b | TC398442 | Predicted protein |
| gma-miR4393b | CF922827 | Hypothesis protein |
| gma-miR4393b | TC350560 | Hypothesis protein |
| gma-miR4393b | TC367785 | Uncharacterized protein At1g80160.2 |
| gma-miR4393b | TC398820 | Hypothesis protein |
| gma-miR4393b | TC367797 | Hypothesis protein |
| gma-miR4393b | TC376836 | Hypothesis protein |
| gma-miR4393b | TC358822 | Hypothesis protein |
| gma-miR4393b | TC368204 | 40S ribosomal protein S23 |
| gma-miR4393b | TC381232 | Serine/threonine protein phosphatase |
| gma-miR4394 | BU546642 | Hypothesis protein |
| gma-miR4394 | GD992183 | Hypothesis protein |
| gma-miR4394 | TC376445 | UDP-glucuronosyl/UDP-glucosyltransferase |
| gma-miR4394 | TC378295 | Hypothesis protein |
| gma-miR4394 | TC366280 | Hypothesis protein |
| gma-miR4394 | FK286598 | DNA-directed RNA polymerase II subunit RPB7 |
| gma-miR4394 | TC418000 | Hypothesis protein |
| gma-miR4394 | FK490281 | Hypothesis protein |
| gma-miR4394 | CO984769 | Hypothesis protein |
| gma-miR4394 | TC349849 | Hypothesis protein |
| gma-miR4394 | TC381217 | Hypothesis protein |
| gma-miR4394 | TC385968 | Hypothesis protein |
| gma-miR4395 | TC350826 | Hypothesis protein |
| gma-miR4395 | TC398543 | Delta(14)-sterol reductase |
| gma-miR4395 | TC413872 | Reverse transcriptase family member |
| gma-miR4395 | TC401969 | Hypothesis protein |
| gma-miR4395 | TC372807 | Malate dehydrogenase [NADP], chloroplast precursor |
| gma-miR4395 | TC362005 | Malate dehydrogenase [NADP], chloroplast precursor |
| gma-miR4395 | TC383158 | 50S ribosomal protein L19 |
| gma-miR4395 | TC371672 | Malate dehydrogenase [NADP], chloroplast precursor |
| gma-miR4395 | GE009764 | Hypothesis protein |
| gma-miR4395 | TC362822 | Predicted protein |
| gma-miR4395 | AW278289 | Hypothesis protein |
| gma-miR4395 | CA783417 | Hypothesis protein |
| gma-miR4395 | DB980571 | Hypothesis protein |
| gma-miR4396 | TC383964 | Hypothesis protein |
| gma-miR4396 | TC355948 | Oligopeptidase B |
| gma-miR4396 | TC379090 | Endo-1,4-beta-glucanase precursor |
| gma-miR4397 | BE610664 | Hypothesis protein |
| gma-miR4397 | TC392510 | Hypothesis protein |
| gma-miR4397 | TC394954 | Hypothesis protein |
| gma-miR4397 | GD723860 | Hypothesis protein |
| gma-miR4397 | TC382998 | ATP-dependent Clp protease |
| gma-miR4397 | GD794022 | Hypothesis protein |
| gma-miR4397 | EV276603 | Hypothesis protein |
| gma-miR4397 | AW310034 | Alcohol dehydrogenase-like protein |
| gma-miR4398 | TC415490 | AAA-metalloprotease FtsH |
| gma-miR4398 | TC386270 | AAA-metalloprotease FtsH |
| gma-miR4398 | TC406477 | Hypothesis protein |
| gma-miR4398 | TC365934 | Sigma-54 dependent transcriptional regulator/response regulator FleR |
| gma-miR4398 | TC402440 | MGC80520 protein |
| gma-miR4398 | FK501220 | Hypothesis protein |
| gma-miR4398 | TC390732 | Hypothesis protein |
| gma-miR4399 | TC386550 | 17.5 kDa class I heat shock protein |
| gma-miR4399 | TC396543 | Hypothesis protein |
| gma-miR4399 | TC375885 | Hypothesis protein |
| gma-miR4399 | BQ094860 | Beta-lactamase domain protein |
| gma-miR4399 | CF805920 | Hypothesis protein |
| gma-miR4399 | CF805920 | Hypothesis protein |
| gma-miR4399 | TC386072 | Hypothesis protein |
| gma-miR4399 | TC404672 | Tubulin A |
| gma-miR4399 | BG881362 | Hypothesis protein |
| gma-miR4399 | TC408875 | Predicted protein |
| gma-miR4399 | TC349893 | Hypothesis protein |
| gma-miR4399 | TC370679 | Multiple myeloma tumor-associated protein 2 |
| gma-miR4399 | TC390574 | Predicted protein |
| gma-miR4399 | TC386662 | Predicted protein |
| gma-miR4399 | TC380940 | Predicted protein |
| gma-miR4400 | TC388978 | Hypothesis protein |
| gma-miR4400 | DB975512 | Catechol-1,2-dioxygenase |
| gma-miR4400 | DB986481 | Hypothesis protein |
| gma-miR4400 | TC378725 | Hypothesis protein |
| gma-miR4400 | TC366668 | Hypothesis protein |
| gma-miR4400 | TC359545 | Hypothesis protein |
| gma-miR4400 | TC375391 | Secondary wall-associated glycosyltransferase family 8D |
| gma-miR4401 | EV276697 | Hypothesis protein |
| gma-miR4401 | TC416593 | Hypothesis protein |
| gma-miR4401 | AI900437 | MtN30 protein |
| gma-miR4401 | TC353266 | Galactokinase |
| gma-miR4401 | TC390975 | Hypothesis protein |
| gma-miR4401 | BE210568 | Alcohol dehydrogenase-like protein |
| gma-miR4401 | TC349091 | Cinnamyl alcohol dehydrogenase |
| gma-miR4401 | BQ299625 | Hypothesis protein |
| gma-miR4402 | CF920922 | Autophagy-related protein 8 |
| gma-miR4402 | TC377247 | KpsM |
| gma-miR4402 | TC373750 | 60S ribosomal protein L27 |
| gma-miR4402 | TC414569 | Hypothesis protein |
| gma-miR4402 | TC350947 | Hypothesis protein |
| gma-miR4403 | TC364969 | Hypothesis protein |
| gma-miR4403 | TC407765 | Hypothesis protein |
| gma-miR4403 | TC408980 | Hypothesis protein |
| gma-miR4403 | GE083655 | Hypothesis protein |
| gma-miR4403 | TC387781 | Hypothesis protein |
| gma-miR4403 | TC354024 | Uncharacterized protein At5g03190.2 |
| gma-miR4403 | BI970951 | Hypothesis protein |
| gma-miR4403 | TC375032 | Hypothesis protein |
| gma-miR4403 | BE210017 | Uncharacterized protein At5g03190.2 |
| gma-miR4403 | TC400616 | Hypothesis protein |
| gma-miR4403 | BU965565 | Predicted protein |
| gma-miR4403 | FG989686 | TPR repeat |
| gma-miR4404 | TC371572 | Hypothesis protein |
| gma-miR4404 | TC374385 | Hypothesis protein |
| gma-miR4404 | TC355127 | Hypothesis protein |
| gma-miR4404 | DW247416 | Hypothesis protein |
| gma-miR4404 | TC385790 | Methionine synthase |
| gma-miR4404 | TC411617 | Hypothesis protein |
| gma-miR4404 | TC370404 | Hypothesis protein |
| gma-miR4404 | TC373173 | Methionine synthase |
| gma-miR4405 | TC386328 | Hypothesis protein |
| gma-miR4405 | TC420098 | Hypothesis protein |
| gma-miR4405 | TC370821 | Hypothesis protein |
| gma-miR4405 | TC414265 | Hypothesis protein |
| gma-miR4405 | TC375839 | Hypothesis protein |
| gma-miR4405 | TC360608 | Hypothesis protein |
| gma-miR4405 | TC352214 | Hypothesis protein |
| gma-miR4405 | TC358688 | Hypothesis protein |
| gma-miR4405 | TC362219 | Hypothesis protein |
| gma-miR4406 | TC378207 | Hypothesis protein |
| gma-miR4406 | TC387311 | Hypothesis protein |
| gma-miR4406 | TC361860 | Hypothesis protein |
| gma-miR4406 | TC395865 | Hypothesis protein |
| gma-miR4406 | BE612180 | Candida glabrata strain CBS138 chromosome J complete sequence |
| gma-miR4406 | TC369677 | Hypothesis protein |
| gma-miR4407 | TC371452 | Isopentenyl transferase |
| gma-miR4407 | TC357141 | Hypothesis protein |
| gma-miR4407 | TC371637 | Hypothesis protein |
| gma-miR4407 | TC416046 | Hypothesis protein |
| gma-miR4407 | FK440477 | MADS box protein AGL1 |
| gma-miR4407 | AW350911 | Hypothesis protein |
| gma-miR4407 | TC377286 | Hypothesis protein |
| gma-miR4407 | AI966262 | Hypothesis protein |
| gma-miR4407 | TC399252 | Hypothesis protein |
| gma-miR4407 | TC379310 | Hypothesis protein |
| gma-miR4407 | BI784728 | MFS permease |
| gma-miR4407 | CA784302 | LeOPT1 |
| gma-miR4407 | TC360993 | Ribulose bisphosphate carboxylase/oxygenase activase |
| gma-miR4407 | TC386682 | Hypothesis protein |
| gma-miR4407 | FG986077 | Fasciclin-like arabinogalactan protein 11 precursor |
| gma-miR4407 | FK020205 | Hypothesis protein |
| gma-miR4408 | TC369177 | Hypothesis protein |
| gma-miR4408 | TC414824 | Hypothesis protein |
| gma-miR4408 | TC403494 | Beta-1,3 glucanase precursor |
| gma-miR4408 | TC418443 | Hypothesis protein |
| gma-miR4408 | FK579143 | Hypothesis protein |
| gma-miR4409 | TC405740 | Hypothesis protein |
| gma-miR4409 | GD864958 | Hypothesis protein |
| gma-miR4409 | TC399132 | Hypothesis protein |
| gma-miR4409 | TC403468 | Two-component response regulator-like protein |
| gma-miR4409 | TC349474 | Hypothesis protein |
| gma-miR4409 | TC360966 | Uncharacterized aarF domain-containing protein kinase |
| gma-miR4409 | CD411571 | Alpha-protein kinase 1 |
| gma-miR4410 | TC372362 | Hypothesis protein |
| gma-miR4410 | TC404021 | Hypothesis protein |
| gma-miR4410 | BF597512 | Hypothesis protein |
| gma-miR4410 | TC419324 | Hypothesis protein |
| gma-miR4410 | TC356937 | Hypothesis protein |
| gma-miR4410 | TC405027 | Hypothesis protein |
| gma-miR4410 | TC384608 | 60S ribosomal protein L11 |
