# Supplementary material for: Characterization of the stress associated microRNAs in Glycine max by deep sequencing
Source: BMC Plant Biol. 2011 Nov 23;11:170. doi: 10.1186/1471-2229-11-170 (PMC3267681; doi:10.1186/1471-2229-11-170)
Supplement: Additional file 10 — The sequences of antisense RNA probes. [file 1471-2229-11-170-S10.DOC]

**Additional file 10 : The sequences of antisense RNA probes**

| **probe name** | **sequence(5'-3')** | **Length (nt)** |
| --- | --- | --- |
|
| gma-MIR166b | AGGGAATGAAGCCTGGTCCGA | 21 |
| gma-MIR169d | CGGCAAGTCATCCTTGGCTCA | 21 |
| gma-MIR482b | TCCTTCCCAATCCCCCCATA | 20 |
| gma-MIR1507a | TCAGACGATGTATGGAATGAGA | 22 |
| Gma-m001 | GTGCTCTCTATCTTCTGTCAG | 21 |
| Gma-m002 | CATGCTCTGCCTGTTCCCCTG | 21 |
