# Supplementary material for: Characterization of the stress associated microRNAs in Glycine max by deep sequencing
Source: BMC Plant Biol. 2011 Nov 23;11:170. doi: 10.1186/1471-2229-11-170 (PMC3267681; doi:10.1186/1471-2229-11-170)
Supplement: Additional file 11 — qRT-PCR primers of miRNAs. [file 1471-2229-11-170-S11.DOC]

Additional file 11: qRT-PCR primers of miRNAs

| miRNA primer | sequence 5'-3' |
| --- | --- |
| gma-MIR167d | TGAAGCTGCCAGCATGATCTG |
| gma-MIR482 | GGAATGGGCTGATTGGGAAGC |
| gma-MIR4369 | GGATCAAGCTGATCCGGAAGTGGA |
| gma-MIR1507a | TCTCATTCCATACATCGTCTGA |
| gma-MIR1508a | TAGAAAGGGAAATAGCAGTTG |
| gma-MIR393a | TCCAAAGGGATCGCATTG |
| gma-MIR156f | TGACAGAAGAGAGAGAGCAC |
| gma-MIR394a | TTGGCATTCTGTCCACCTCC |
| gma-MIR4397 | TGTCAAAGATGTGGCGAATAC |
| gma-MIR169d | TGAGCCAAGGATGACTTGCCG |
| 5s rRNA | GGAAAAATAGCTCGGCGCCA |
